# Androgen deprivation restores ARHGEF2 to promote neuroendocrine differentiation of prostate cancer

**DOI:** 10.1038/s41419-022-05366-8

**Published:** 2022-11-05

**Authors:** Xuanrong Chen, Yi Shao, Wanqing Wei, Shimiao Zhu, Yang Li, Yutong Chen, Hanling Li, Hao Tian, Guijiang Sun, Yuanjie Niu, Zhiqun Shang

**Affiliations:** 1 Department of Urology, Tianjin Institute of Urology, The second hospital of Tianjin Medical University, Tianjin, China; 2 Department of Pediatric Surgery, Huai’an Maternal and Children Health Hospital, Huai’an, China

**Keywords:** Prostate cancer, Mechanisms of disease

## Abstract

Androgen receptor (AR) plays an important role in the progression of prostate cancer and has been targeted by castration or AR-antagonists. The emergence of castration-resistant prostate cancer (CRPC) after androgen deprivation therapy (ADT) is inevitable. However, it is not entirely clear how ADT fails or how it causes resistance. Through analysis of RNA-seq data, we nominate ARHGEF2 as a pivotal androgen-repressed gene. We show that ARHGEF2 is directly suppressed by androgen/AR. AR occupies the enhancer and communicates with the promoter region of ARHGEF2. Functionally, ARHGEF2 is important for the growth, lethal phenotype, and survival of CRPC cells and tumor xenografts. Correspondingly, AR inhibition or AR antagonist treatment can restore ARHGEF2 expression, thereby allowing prostate cancer cells to induce treatment resistance and tolerance. Overall, our findings provide an explanation for the contradictory clinical results that ADT resistance may be caused by the up-regulation of ARHGEF2 and provide a novel target.

## Introduction

Prostate cancer (PCa) has become the second most common cancer in men, and both morbidity and mortality rates have been on the rise in recent years [1]. As a heterogeneous disease, the occurrence of PCa may be affected by multiple factors such as genes, cellular context, and environmental factors [2, 3]. Cumulative evidence suggests that the androgen receptor (AR) signaling is involved in the carcinogenesis, progression, and recurrence of PCa [4]. In cells, once bound by androgen, AR translocates to the nucleus, where it binds to the chromatin at androgen response elements (ARE) to turn on prostate-specific gene expression such as prostate-specific antigen (PSA) [5]. Through the integration of genome-wide expression profile data and cistrome data, recent progress has been made in the understanding of AR transcription regulation. In addition to its well-understood roles in gene activation, AR was recently shown to function as a transcriptional repressor [6, 7]. However, in contrast to the plethora of AR-induced genes reported in the literature, only a few have been shown to be directly inhibited by AR [8–10]. A majority of the AR-repressed genes are yet to be identified. Further, how AR directly suppresses gene expression is not fully understood and how AR-mediated repression contributes to PCa and resistance to AR-targeted therapy remains uncharacterized [11].

While androgen deprivation therapy (ADT) remains the cornerstone of the treatment of advanced PCa, the subsequent progress toward castration-resistant prostate cancer (CRPC) has led to a lethal disease [12, 13]. Importantly, although CRPC is resistant to AR-targeted therapy, it continues to be sustained and dependent on AR signaling, such as mutations in ligand binding domain (F877L and T878A) of AR, AR amplification, or activation of AR-targets through steroid-inducible glucocorticoid receptor [14–17]. Thus, higher affinity next-generation anti-androgen compounds, such as enzalutamide (MDV3100), apalutamide, and abiraterone acetate, are useful in preventing the progress of CRPC [18, 19]. Although these next-generation AR-targeted inhibitors can prolong the overall survival of patients, the overall survival benefit is temporary, and the disease will eventually worsen and progress. The major clinical challenges for these patients stemmed from the emergence of highly aggressive phenotypes, such as lineage plasticity and acquisition of a neuroendocrine PCa (NEPC) phenotype or double-negative PCa (DNPC), which lacks both NEPC-specific and CRPC-specific markers [20]. Recent evidence suggests that NEPC can emerge from pre-existing adenocarcinoma in the advanced stages of prostate cancer progression and develop resistance to AR-targeted therapy [21, 22]. This is an adaptive resistance mechanism, and treatment-related NEPC is associated with a poor prognosis. More effort is needed to better understand the drivers of lineage plasticity and the acquisition of treatment resistance, and how to best utilize treatment strategies aimed at killing these pluripotent cells or restoring them to a sensitive state.

The ARHGEF2 (Rho/Rac Guanine Nucleotide Exchange Factor 2) gene is a member of the guanine nucleotide exchange factor that plays a role in controlling the activity of downstream effectors, via the Rho GTPases family [23]. Rho GTPases play a fundamental role in numerous cellular processes that are initiated by extracellular stimuli that work through G protein coupled receptors and form complexes with G proteins that stimulate Rho-dependent signals [24, 25]. It has been shown in different cell types that ARHGEF2 is a key molecule in actin remodeling and cell barrier dysfunction [26]. ARHGEF2 was also identified in genome-wide small hairpin RNA (shRNA) screens, which aim to identify genes required for the survival of human breast, colon, lung, ovarian, and pancreatic cell lines, suggesting that ARHGEF2 may be a contributor to tumorigenesis [27]. In this study, we discovered that ARHGEF2 highly expressed in NEPC clinical cohort, patient tissue and mouse model. ARHGEF2 is transcriptionally repressed by AR, and the AR-targeted therapy relieves this repression leading to ARHGEF2 up-regulation. In addition, experiments demonstrate that ARHGEF2 functions as a driver of lineage plasticity in PCa and can induce an NE-like phenotype. We identify that ARHGEF2 regulates SOX2 via FGFR1/MAPK pathway to drive the NE-like phenotype. Notably, we also ARHGEF2 provide a novel target in PCa. In summary, our findings draw attention to the widespread use of AR-targeted therapy and the emergence of adaptive resistance mechanisms related to ARHGEF2 that promote neuroendocrine differentiation in anti-androgen-induced PCa.

## Materials and methods

### RNA-seq and pathway analysis

To examine the direct effect of AR signaling on gene transcription, the cells were perfused with charcoal-dextran stripped fetal bovine serum (CD-FBS) for 3 days, and then treated with 10 nM dihydrotestosterone (DHT) or DMSO for 24 h. Deep sequencing of rRNA-depleted total RNAs was performed in biological duplicates on LNCaP cells. To examine the biological effect of ARHGEF2 in PCa cells, 22RV1 cells were transfected with siARHGEF2 (#1 and #2) and SCRAMBLE siRNAs for 48 h. RNA high throughput sequencing was performed by Cloud-Seq Biotech (Shanghai, China). Briefly, total RNA of each sample was extracted using RNeasy Mini Kit (Qiagen). Total RNA of each sample was quantified and qualified by Agilent 2100 Bioanalyzer (Agilent Technologies, Palo Alto, CA, USA), NanoDrop (Thermo Fisher Scientific Inc.) and 1% agrose gel. 1 μg total RNA with RIN value above 6.5 was used for the following library preparation. The poly(A) mRNA isolation was performed using Poly(A) mRNA Magnetic Isolation Module or rRNA removal Kit. Next generation sequencing library preparations were constructed according to the manufacturer’s protocol. RNA libraries were constructed by using NEBNext Ultra II Directional RNA Library Prep Kit (New England Biolabs, Inc., Massachusetts, USA) according to the manufacturer’s instructions. Libraries were controlled for quality and quantified using the BioAnalyzer 2100 system (Agilent Technologies, Inc., USA). Library sequencing was performed on an illumina Novaseq 6000 instrument with 150 bp paired-end reads. After 3′ adaptor-trimming and low-quality read removing by cutadapt software (v1.9.3), the high-quality clean reads were aligned to the reference genome (UCSC hg19) with hisat2 software (v2.0.4). Then, HTSeq software (v0.9.1) was used to get the raw count, and the edgeR (v3.32.1) Bioconductor package was used to perform normalization, then differentially expressed mRNAs were identified by *p*-value and fold change. 189 genes were downregulated in 22RV1 siARHGEF2 cells (Fold Change cut-off: 2.0; *P*-value cut-off: 0.05), which were further analyzed for enriched pathways. Pathway analysis is a functional analysis mapping gene to KEGG pathways. The Fisher p-value denotes the significance of the pathway correlated to the condition that was enriched (*P*-value cut-off is 0.05.) GSEA analysis was performed in the 22RV1 siARHGEF2 and 22RV1 si-NC groups to explore the biological signaling pathway [28]. The MAPK_Pathway with significant enrichment results was demonstrated on the basis of enrichment score (ES) and *p*-value.

### Human prostate cancer specimens

Tissue microarrays (TMA) for prostate cancer (PCa) specimens were obtained from the second hospital of Tianjin Medical University, acquiring the due consent from the patients and mandatory approval from the Institutional Review Board as described previously [29].

### Animal model

TRAMP (Transgenic Adenocarcinoma of Mouse Prostate) [C57BL/6] mice were obtained from the Jackson Laboratories repository. Mouse-tail DNA was collected from the litter and subjected to PCR as described previously [30]. Whole murine prostates were micro-dissected from mice at the indicated age, imaged, weighed, and then fixed overnight in 4% PFA. Sections were then transferred to 70% EtOH solution and paraffin embedded, and sectioned.

### Immunohistochemistry (IHC) staining

IHC for AR (ab9474, Abcam), FGFR1 (9740, CST), P-ERK (4370, CST), SOX2 (3579, CST), CHGA (ab45179, Abcam), SYP (ab52636, Abcam) and ARHGEF2 (ab155785, Abcam) was performed using PV-6000 system (ZSGB-BIO, China). Briefly, the slides were immersed in 1X Tris-EDTA (pH 9.0) buffer (Solarbio, China) and placed in a microwave oven for 10 min on high heat, and then adjusted to medium-low heat for 10 min to restore the antigen. 3% H_2_O_2_ was added to remove endogenous peroxidase in tissue samples. Cover the tissue on the slide with the primary antibody, place it in a humid box, and incubate overnight at 4 °C. After rewarming at room temperature for 30 min, horseradish peroxidase-linked secondary antibody (ZSGB-BIO, PV-6000, China) was added to the specimen and incubate the slides at room temperature for 30 min. After being stained with the DAB solution, the slides were immediately placed in water to stop dyeing, slides were subsequently counterstained with hematoxylin. The tissue is then dehydrated and preserved with neutral balsam (OriGene, ZLI- 9555, China).

### Immunostaining

Immunostaining was performed as described before [31]. Briefly, cells were grown on cover glasses and fixed for 15 min with 4% paraformaldehyde (PFA) solution at room temperature. Cells were washed twice with PBS and then permeabilized with 0.1% Triton-X100 in PBS for 10 min. Then, cells were incubated with a blocking buffer (PBS with 5% BSA). Primary antibody was applied at the specified dilution in blocking solution overnight at 4 °C. The following morning, cells were washed twice with PBS and incubated with the appropriate fluorescent secondary antibody (in blocking solution) for 30 min in the dark. Coverslips were then washed three times with PBS, mounted with DAPI, and examined under Olympus FV1000D microscope. The following antibodies were used for immunostaining: ARHGEF2 (ab201687, Abcam), CHGA (ab45179, Abcam), and AR (ab133273, Abcam).

### Cell lines

All the prostate cancer (22RV1, LNCaP, PC3, and DU145) cell lines were obtained from American Type Cell Culture (ATCC) and maintained as per guidelines. Briefly, cells were cultured in the recommended media supplemented with 10% fetal bovine serum (FBS) (Gibco) and 0.5% Penicillin Streptomycin (Thermo Fisher Scientific) in cell culture incubator (Thermo Fisher Scientific) supplied with 5% CO_2_ at 37 °C. LNCaP-AI was generated as described before and cultured in RPMI 1640 media supplemented with 10% charcoal stripped serum (CD-FBS; Gibco) [29]. For certain experiments, different cell lines were used based on their characteristics and potential significance. Briefly, the LNCaP cell line is an AR positive and androgen sensitive human PCa cell [32, 33]. The LNCaP-AI cell line is an AR positive and androgen independent human PCa cell, generated from LNCaP cells [29]. PC3 and DU145 cell lines are negative for AR expression and show androgen-independent responses [32, 34, 35]. 22Rv1 cells harbor the H874Y mutation in the AR and are resistant to castration [32, 36].

### Androgen stimulation and deprivation

For androgen stimulation, cells were starved for 72 h in RPMI 1640 media (Gibco) supplemented with 10% CD-FBS (Gibco) followed by stimulation with dihydrotestosterone (DHT) (Sigma-Aldrich) at the indicated time points and indicated concentrations. For anti-androgen treatment, LNCaP and 22RV1 cells were hormone-starved for 48 h using RPMI 1640 media supplemented with 10% CD-FBS (Gibco) followed by treatment with enzalutamide (cat. HY-70002, MedChem Express) for 48 h in complete medium.

### Transfections

Transfections with siRNAs (purchased from GenePharma, China) were carried out using Lipofectamine RNAiMAX Transfection Reagent (ThermoFisher Scientific, USA) with cells at 50% confluence cultured in 6-well plates. The siRNA sequences are listed in Supplementary Table 1. To achieve the maximal inhibition effect, two siRNAs were mixed together and transfected into cells. LNCaP-AI and 22RV1-shARHGEF2 cells were generated using lentiviral shRNA against ARHGEF2 or SCRAMBLE (SWS Biotechnology, Tianjin, China). Overexpression of ARHGEF2 was generated using lentiviral mediated vector (SWS Biotechnology, Tianjin, China), and empty vector was used as control. After infection, cells were selected with puromycin (1 ug/ml) for 3 days to remove uninfected cells, and then maintained in puromycin-containing complete medium.

### RNA analysis

Total RNA was isolated using TRIzol Reagent (Invitrogen, USA) as the standard RNA isolation procedure. About 5 μg of total RNA with oligo (dT) primers was reverse-transcribed in a 20 μL volume using the RevertAid First Strand cDNA Synthesis kit (Thermo Fisher Scientific, Inc.) followed by the manufacturer’s protocol. For regular PCR, all reactions were set up as follows: 10 µl 2× Taq PCR MasterMix (with dye), 0.4 µl forward primer (10 nM), 0.4 µl reverse primer (10 nM), 1 µl cDNA and 8.2 µl ddH_2_O, for a total reaction volume of 20 µl. The reaction system was preheated at 94 °C for 3 min, and then performed using the following thermal cycle program: 94 °C for 30 s, 55 °C for 30 s, 72 °C for 30 s, and 40 cycles, followed by 72 °C for 5 min. DNA products were analyzed in 1% agarose gel. For quantitative PCR, all reactions were set up as follows: 10 µl FastStart Universal SYBR Green Master (Roche), 0.2 µl forward primer (10 nM), 0.2 µl reverse primer (10 nM), 1 µl cDNA and 8.6 µl ddH_2_O, for a total reaction volume of 20 µl. The reaction system was preheated at 95 °C for 10 min, and then performed using the following thermal cycle program: 95 °C for 15 s, 72 °C for 20 s, and 40 cycles. Relative expression of target genes was calculated using the 2-ΔΔCt method using GAPDH as an internal control. The primers are listed in Supplementary Table 2.

### Immunoblot analysis

Cell lysates were prepared in cell lysis buffer (Cat.R0020, Solarbio), supplemented with Protease Inhibitor Cocktail (Roche). Protein concentration was determined by Bradford (Pierce Bradford Assay Kit, Thermo Scientific) at 595 nm. Western blot analysis was performed by 10% sodium dodecyl sulfate-polyacrylamide gel electrophoresis (SDS-PAGE). Briefly, 50 µg proteins were separated by 10% SDS-PAGE and then transferred onto an Immobilon-P membrane (Millipore, USA). The membrane was blocked with 5% non−fat dry milk for 1 h at room temperature and then incubated overnight at 4 °C with primary antibody. Subsequently, blots were washed in 1× tris-buffered saline, 0.1% Tween 20 (TBS-T) buffer and incubated with secondary antibody (Goat anti-mouse IgG (H + L), HRP conjugate, cat. SA00001-1; Goat Anti-Rabbit IgG (H + L), HRP conjugate, cat. SA00001-2, Proteintech) for 1 h, washed, and processed using the Enhanced chemiluminescence (ECL) reagent (Millipore), then visualized by Tanon (model 4500, Tanon) system. The primary antibodies used are as follow: 1:1000 diluted ARHGEF2 (Abcam, cat. ab155785), 1:1000 diluted p44/42 MAPK (ERK) (CST, cat. 9102), 1:1000 diluted phospho-p44/42 MAPK (P-ERK) (Thr202/Tyr204) (CST, cat. 9101), 1:2000 diluted AR (Abcam, cat. Ab133273), 1:1000 diluted KLK3 (CST, cat. 5365), 1:2000 diluted CHGA (Abcam, cat. ab45179), 1:2000 diluted SYP (Abcam, cat. ab52636), 1:1000 diluted SOX2 (CST, cat. 3579), 1:1000 diluted FGFR1 (CST, cat.9740) and 1:5000 diluted GAPDH (Abcam, cat. ab8245).

### Chromatin immunoprecipitation (ChIP) assay and ChIP-qPCR

ChIP was performed by EZ-Magna ChIP™ A/G kit (Catalog: 17-10086; Millipore) following the manufacturer’s instructions. Briefly, 1 × 10^7^ cells were cross-linked with 37% formaldehyde (Catalog: F8775; Sigma) at room temperature for 10 min. Nuclei were extracted with Nuclear Lysis Buffer, and cross-linked DNA was sheared to 200–1000 base pairs using SONICS Vibra-Cell™ Ultrasonic Liquid Processors (model VCX130; Sonics & Materials, Inc.). Antibody for ChIP assays was purchased from Millipore (AR, rat, cat. 17-10489). ChIP-qPCR was performed with the following parameters: Initial Denaturation at 94 °C for 10 min, followed by 50 cycles of Denature at 94 °C for 20 s and Anneal and Extension at 60 °C for 1 min. Primers for ChIP-qPCR are listed in Supplementary Table 3.

### CUT & Tag (Cleavage under targets and tagmentation)

The CUT and Tag assay (CUT & Tag also referred to as “ChIP-seq”) was performed to determine the AR binding sites in LNCaP cells upon DHT treatment within 24 h. The detailed, step-by-step protocol was followed by Steven Henikoff protocol at https://www.protocols.io/view/bench-top-cut-amp-tag-bcuhiwt6. Briefly, cells were harvested, counted, and centrifuged for 3 min at 600 × *g* at room temperature, washed twice in 1.5 mL Wash Buffer (20 mM HEPES pH 7.5; 150 mM NaCl; 0.5 mM Spermidine; 1× Protease inhibitor cocktail), and incubated with activated concanavalin A coated magnetic beads (Bangs Laboratories) at RT for 15 min. Then, the bead-bound cells were resuspended in 50–100 µL Dig-wash Buffer (20 mM HEPES pH 7.5; 150 mM NaCl; 0.5 mM Spermidine; 1× Protease inhibitor cocktail; 0.05% Digitonin) containing 2 mM EDTA and incubated with 5 µL of the AR primary antibody (Millipore; cat. 17-10489) overnight at 4 °C. Cells were then incubated with pA-Tn5 at RT for 1 h and resuspended in 50–100 µL Tagmentation buffer (10 mM MgCl2 in Dig-med Buffer) and incubated at 37 °C for 1 h. 2.25 µL of 0.5 M EDTA, 2.75 µL of 10% SDS and 0.5 µL of 20 mg/mL Proteinase K was added to stop tagmentation with incubation at 55 °C for 30 min, and then at 70 °C for 20 min to inactivate Proteinase K. Followed by DNA extraction, library amplification, post-PCR clean-up, paired-end NovaSeq 6000 sequencing was performed. Trimmomatic (version 0.40) was used to remove adapters and low-quality reads. Quality distribution plots and base content distribution were generated by FASTQC (version 0.11.9). Before read-mapping, clean reads were obtained from the raw reads by removing the adaptor sequences. The clean reads were then aligned to reference genome (hg38) sequences using the Burrows-Wheeler-Alignment Tool (bwa). The bam file generated by the unique mapped reads as an input file, using macs2 (version 2.2.7.1) for peak calling with cutoff q-value < 0.05. The data have been deposited into the CNGB Sequence Archive (CNSA) of China National GeneBank DataBase (CNGBdb) with accession number CNP0001628.

### Plasmids and luciferase assay

The pGL3-ARHGEF2-PP (GEF-H1 PP) construct was obtained by cloning the ARHGEF2 proximal promoter (ARHGEF2-PP) from the pGL3-basic vector (Promega), and pGL3-ARHGEF2-DP (GEF-H1 DP) was generated by cloning the distal promoter of the ARHGEF2 gene in the pGL3-basic vector (Promega). The DNA sequences are listed in Supplementary Table 4. 22RV1 cells were plated at 40–50% confluency in a 24-well plate and were transfected with pGL3-ARHGEF2-PP (500 ng) and pRL-TK vector (5 ng) using Lipofectamine 3000 Transfection Reagent. For androgen stimulation, the 22RV1 cells were serum starved for 48 h and stimulated with DHT at indicated concentrations for 24 h in RPMI1640 media containing 10% CD-FBS. For anti-androgen treatment, 22RV1 cells were treated with enzalutamide (10 µM) for 24 h. After 24 h of transfection with luciferase constructs, cells were harvested using the lysis buffer provided with the Dual-Glo Luciferase assay kit (Promega). Firefly and Renilla luciferase activity were measured according to the manufacturer’s protocol using GloMax Luminometer (Promega). For each sample, firefly luciferase activity was normalized to Renilla luciferase activity.

### Cell cycle

Cells were harvested, washed, re-suspended, incubated with Propidium Iodide Staining Solution (BD Pharmingen) in the dark for 15 min, and subjected to flow cytometry analysis using ModFit LT software (Verity Software House, Topsham, ME, USA).

### MTT and migration assay

Cell growth was determined by 3-(4,5-dimethylthiazol-2-yl)-2,5-diphe-nyltetrazolium bromide proliferation assays. Migration assay was performed using Transwell chambers of 8μm pore size (Corning) as described before [37].

### Xenograft assay

Nude mice (6–7 weeks old, *n* = 10) were purchased from Beijing HFK Bioscience Co. Ltd. (Beijing, China). The animal studies were approved by Tianjin Institute of Urology, Tianjin, China. Subcutaneous tumor growth assays were performed with 22RV1 shSCR (control) and shARHGEF2 stable cell lines (2 × 10^6^ shSCR cells injected to 5 mice separately, 2 × 10^6^ shARHGEF2 cells injected to another 5 mice separately). At the end point, all mice were sacrificed, and the tumors were harvested under standard, institutionally approved processes.

### Statistical analysis

Statistical analysis was performed using GraphPad Prism 8.0 software (San Diego, CA, USA). Differences were measured using either one-way ANOVA, two-way ANOVA with the post hoc multiple comparisons test, or unpaired two-tailed Student’s t-test or otherwise mentioned. *P* < 0.05 is considered statistically significant, where *P < 0.05, **P < 0.01, ***P < 0.001, ****P < 0.0001 and NS denotes nonsignificant. The results were shown as mean ± standard deviation (SD).

## Results

### ARHGEF2 is highly expressed in NEPC

**Fig. 1:**
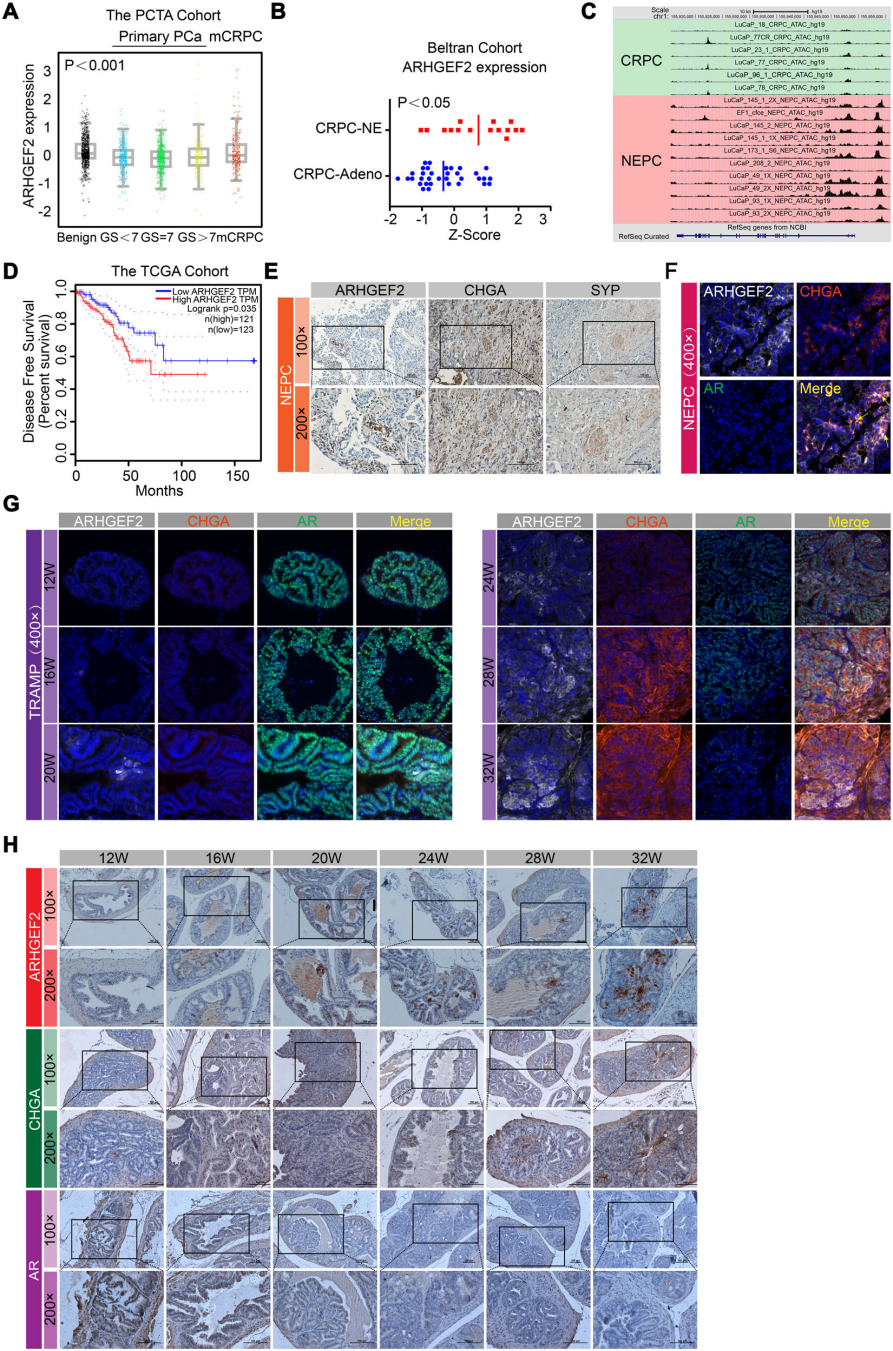
ARHGEF2 highly expressed in neuroendocrine prostate cancer (NEPC). **A** The expression level of ARHGEF2 in the PCTA cohort [39]. **B** The expression level of ARHGEF2 in the Beltran cohort [20]. **C** Snapshot of the chromatin accessibility on the ARHGEF2 gene locus in both CRPC and NEPC tissues from a clinical cohort [41]. **D** Kaplan-Meier analysis of OS based on ARHGEF2 expression in the TCGA cohort [58]. **E** Representative images of ARHGEF2, CHGA, and SYP staining in NEPC patient tissue. **F** Immunoblot for ARHGEF2 (white), CHGA (red), and AR (green) in NEPC patient tissues. **G** Immunoblot for ARHGEF2 (white), CHGA (red), and AR (green) in TRAMP mice from prostate tissue acquired during different times (from 12 weeks to 32 weeks). **H** IHC analysis of ARHGEF2, CHGA, and AR expression in TRAMP mice from prostate tissue acquired during different times (from 12 weeks to 32 weeks). Data represent mean ± SD. For panel **A** Rank sums-test was applied. For panel **B** two-tailed unpaired Student’s t-test was applied; ****P ≤ 0.0001.

Altered ARHGEF2 activity was reported to be a crucial determinant in human disease pathogenesis, where ARHGEF2 can be a novel therapeutic target in multiple human diseases [23]. To investigate the clinical association of ARHGEF2 in human PCa samples, we first analyzed the genetic alteration pattern of ARHGEF2 in human PCa samples from the cBioportal website (Supplementary Fig. 1). The data revealed that over 30% CRPC and over 15% NEPC patients harbored ARHGEF2 gene amplification status, but less than 5% in castration-sensitive prostate cancer patients. Given that the NE population in PCa increases with disease progression, we sought to determine whether there is a correlation between ARHGEF2 expression and PCa stage [38]. The RNA level of ARHGEF2 were elevated in mCRPC samples (Fig. 1A; *p* < 0.001) in the PCTA cohort [39]. Expression of ARHGEF2 was also significantly increased in neuroendocrine differentiation CRPC (CRPC-NE) samples compared to CRPC with adenocarcinoma characteristics (CRPC-Adeno) samples (Fig. 1B). Chromatin accessibility defines regulatory elements within the genome and is dynamically established to control gene expression [40]. Interestingly, we found that chromatin accessibility is significantly enhanced in the promoter region of ARHGEF2 in NEPC samples compared to CRPC, which suggested that the landscape of ARHGEF2 locus accessibility changes dynamically and correlated to its upregulation in NEPC (Fig. 1C) [41]. Moreover, the PCa patients with high ARHGEF2 expression had shorter overall survival (OS) (Fig. 1D). To further validate this finding, we validated ARHGEF2 expression in patient samples by multiple immunostainings (Fig. 1E, F). Similarly, ARHGEF2 protein expression was largely overlapped with NE marker CHGA. To support the expression pattern of ARHGEF2 in NEPC, we detected the ARHGEF2 expression in the transgenic adenocarcinoma of mouse prostate (TRAMP) model. TRAMP mice express SV40 T antigen (Tag) in their prostate epithelial cells under the regulation of the rat probasin promoter and inactivation of p53 and retinoblastoma protein by SV40 Tag has been associated with the NEPC in TRAMP mice [42–44]. NEPC marker could be found in more than 92–100% of poorly differentiated tumors in the TRAMP mice [44, 45]. Immunostainings and IHC in TRAMP mice showed that ARHGEF2 expression was highly expressed in poorly differentiated tumors in the TRAMP mice and associated with NE marker CHGA expression, thus reaffirming the association between ARHGEF2 and NE phenotype (Fig. 1G, H). These findings support the notion that ARHGEF2 expression was elevated in high-grade and advanced tumors, especially in NEPC.

### ARHGEF2 expression in prostate cancer cells is suppressed by androgen

**Fig. 2:**
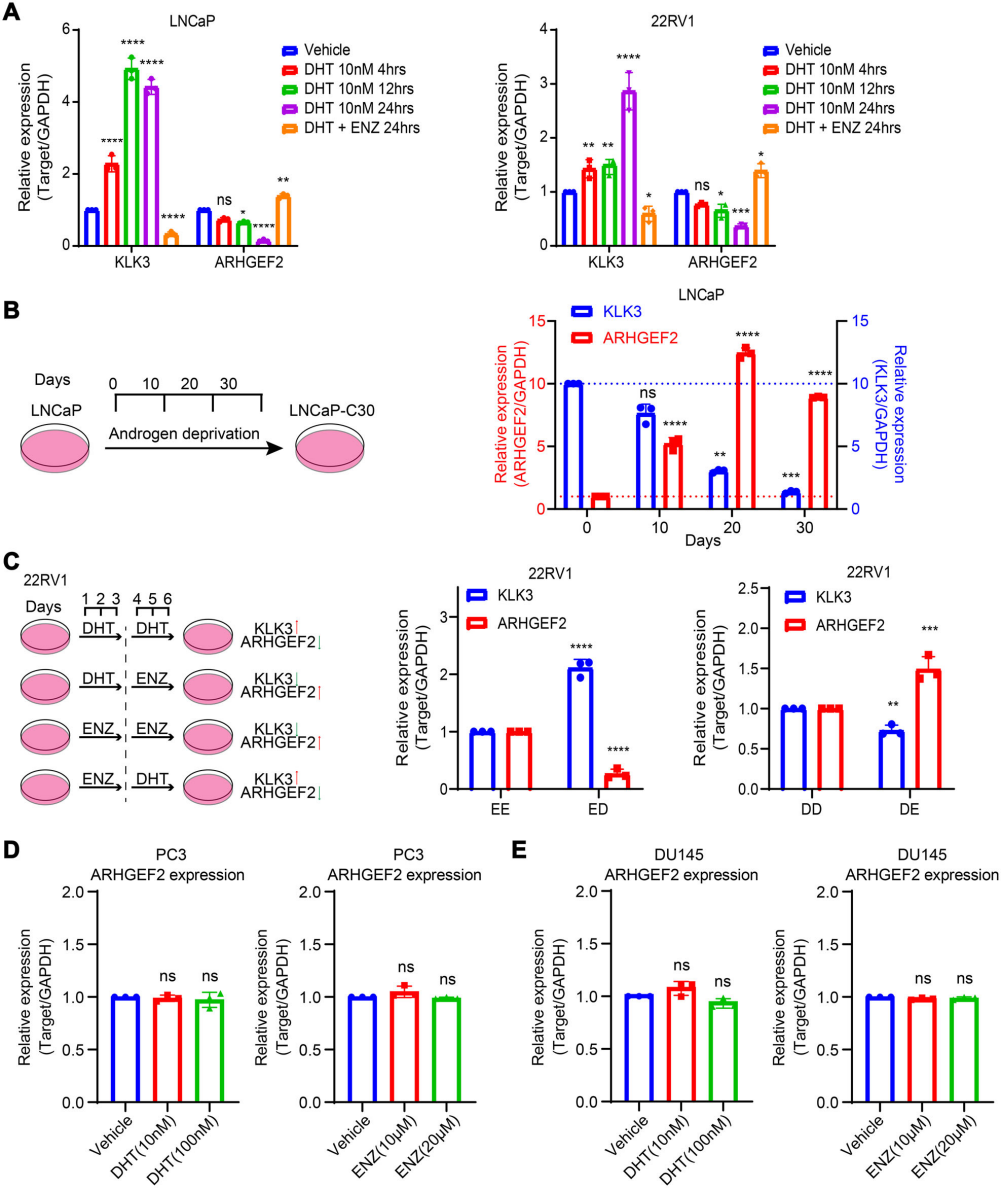
ARHGEF2 expression is suppressed by AR in prostate cancer cells. **A** QPCR data showing the relative expression of ARHGEF2 and KLK3 in LNCaP (left) and 22RV1 (right) cells stimulated for several DHT (10 nM) treatment periods and enzalutamide. **B**. QPCR data showing relative expression of ARHGEF2 and KLK3 in long-term androgen deprived LNCaP cells (LNCaP-C30, LNCaP cells were cultured in androgen deprived medium for 30 days). **C** (Left) Schema depicting the sequential treatment of 22RV1 cells with DHT (10 nM) and enzalutamide (ENZ, 10 µM). (Right) QPCR data showing relative expression of ARHGEF2 and KLK3 using cells with sequential treatment of 22RV1 cells with DHT and/or ENZ as depicted. **D** QPCR data showed relative expression of ARHGEF2 using PC3 cells (an AR-negative PCa cell line) with DHT (10 nM and 100 nM) and enzalutamide (ENZ, 10 µM and 20 µM). **E**. QPCR data showed relative expression of ARHGEF2 using DU145 cells (an AR-negative PCa cell line) with DHT (10 nM and 100 nM) and enzalutamide (ENZ, 10 µM and 20 µM). For panels **A** and **B**, two-way ANOVA, Dunnett’s multiple-comparisons test; **C**, two-way ANOVA, Sidak’s multiple-comparisons test; **D** and **E**, two-tailed unpaired Student’s t-test was applied. Error bar indicates the standard deviation (SD). *P ≤ 0.05, **P ≤ 0.01, ***P ≤ 0.001, ****P < 0.0001 and ns not significant.

Studies have shown that AR directly inhibits the expression of the AR gene itself, as well as other genes [46]. To confirm androgen inhibition of ARHGEF2, we performed reverse transcriptase-PCR analysis of LNCaP and 22RV1 cells treated with 10 nm DHT for 0, 4, 12, and 24 h, and KLK3, an androgen-induced gene, was used as a positive control (Supplementary Fig. 2A). Stimulating cells with DHT, resulted in a significant decrease (80% to baseline) in expression of ARHGEF2 and a dramatic increase in the expression of the KLK3 gene by quantitative PCR (Fig. 2A). To corroborate these findings, enzalutamide (ENZ), a widely used non-steroidal pharmacological inhibitor of AR in the treatment of locally advanced non-metastatic and metastatic PCa, was used in LNCaP and 22RV1 cells. Enzalutamide treatment significantly increased (~1.5-fold) the transcription level of ARHGEF2 (Fig. 2A).

To cross-validate these findings, we analyzed the public data set (GSE71797) of 22RV1 and VCaP cells stimulated by R1881 (a synthetic androgen). It showed a decrease in the expression of several previously known AR repressed genes (NOV and BCHE) and the expression of ARHGEF2 was also reduced after R1881 treatment (Supplementary Fig. 2B). Furthermore, immunoblot analysis revealed a concordant decrease in ARHGEF2 protein following DHT stimulation in a time-dependent manner (Supplementary Fig. 2C). To estimate the effect of long-term androgen deprivation on ARHGEF2 expression, LNCaP cells were cultured in androgen-deprived conditions for 30 days (Fig. 2B). Remarkably, in combination with prolonged androgen deprivation, a robust increase in ARHGEF2 expression was observed. Since 22RV1 cells are less responsive to androgen stimulation compared to LNCaP cells, to further investigate the responsiveness to androgen stimulation, we used the 22RV1 cell line to examine the effect of androgen stimulation [10]. We primed the 22RV1 cells either with DHT (10 nM) or ENZ (10 µM) for 3 days, followed by ENZ treatment or DHT stimulation for the next 3 days (Fig. 2C). As anticipated, blocking androgen signaling with ENZ in the androgen-primed 22RV1 cells resulted in a significant increase in ARHGEF2 expression, while ENZ-treated 22RV1 cells stimulated with DHT showed repression of ARHGEF2 (Fig. 2C). Besides, quantitative PCR and immunoblot analysis were also used to test whether DHT stimulation also inhibits ARHGEF2 expression in AR-negative prostate cancer cell lines (PC3 and DU145) (Fig. 2D, E and Supplementary Fig. 3D, E). No significant differences in ARHGEF2 expression were observed in these two cell lines, nor in those following enzalutamide treatment (Fig. 2D, E and Supplementary Fig. 3D, E).

### AR directly participates in the transcriptional repression regulation of ARHGEF2 in PCa

**Fig. 3:**
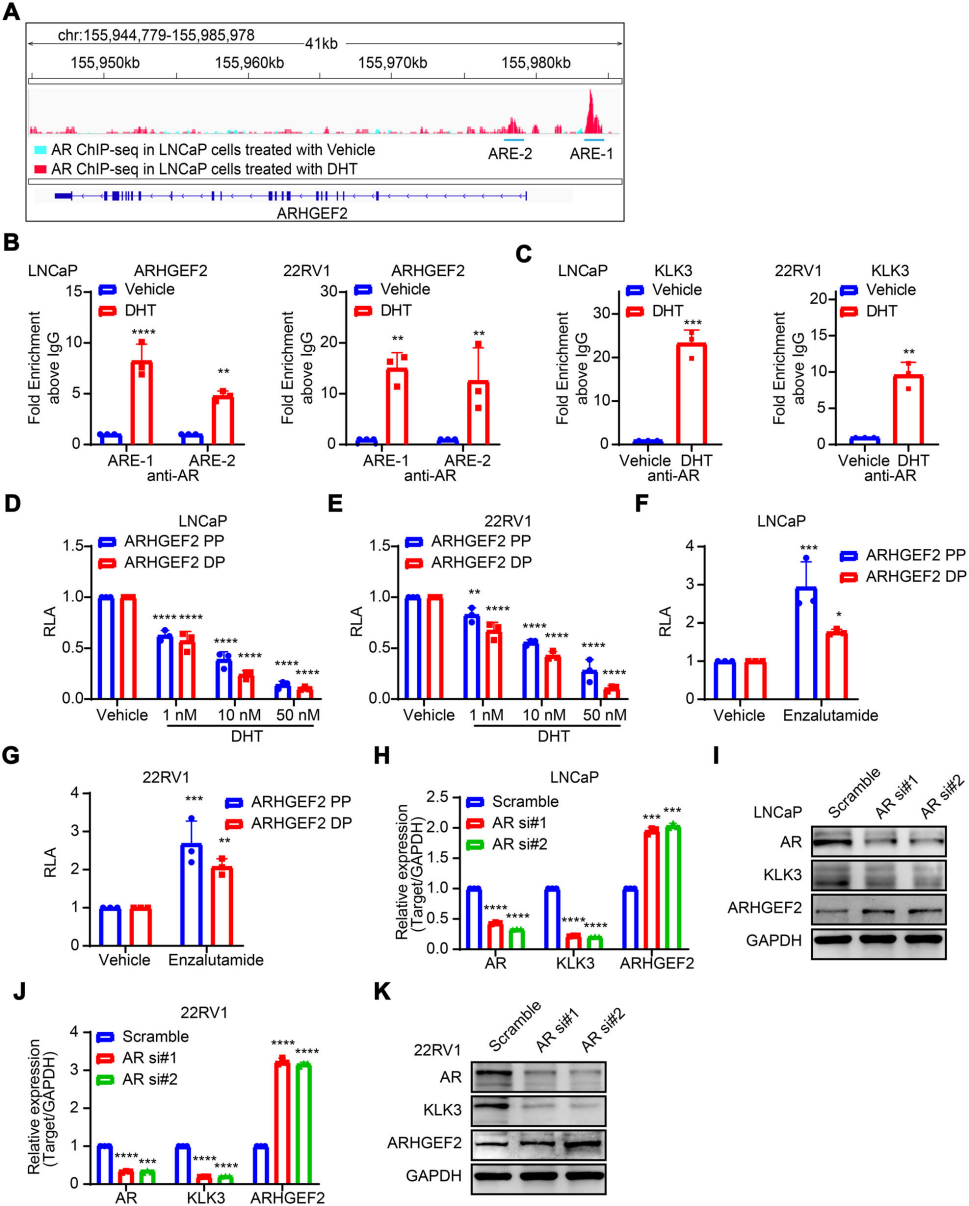
AR directly participates in transcriptional regulation of ARHGEF2 and modulates its expression. **A** Cut-Tag (ChIP-seq) track for AR overlapping signals (blue: AR binding status in LNCaP cells in Vehicle treatment; red: AR binding status in DHT treatment) at ARHGEF2 gene region. Schema showing genomic locations for the AREs on the ARHGEF2 gene flank relative to TSS region (transcriptional start site). For AR ChIP-seq data, the GSM3567212 dataset was acquired to demonstrate AR binding status in LNCaP cells treated with vehicle. Our Cut-Tag assay was performed to demonstrate AR binding status in LNCaP cells treated with DHT. **B**, **C** ChIP-qPCR data showing recruitment of AR to the ARHGEF2 **B** and KLK3 **C** promoters in LNCaP and 22RV1 cells after DHT (10 nM) stimulation 24 h. **D**, **E** Luciferase reporter activity of the proximal (ARHGEF2-PP) and distal ARHGEF2 (ARHGEF2-DP) promoters in DHT (10 nM) stimulated LNCaP **D** and 22RV1 **E** cells. **F**, **G** Luciferase reporter activity of the proximal (ARHGEF2-PP) and distal ARHGEF2 (ARHGEF2-DP) promoters in enzalutamide (10 µM) stimulated LNCaP (**F**) and 22RV1 (**G**) cells. **H**, **I** QPCR **H** and immunoblot **I** data showing relative expression of AR and ARHGEF2 in AR-silenced and control LNCaP cells. **J**–**K** QPCR **J** and immunoblot **K** data showing relative expression of AR and ARHGEF2 in AR-silenced and control 22RV1 cells. Experiments were performed with *n* = 3 biologically independent samples; data represents mean ± SD. For panels **C** two-tailed unpaired Student’s t-test; **B**, **D**–**H** and **J** two-way ANOVA, Sidak’s multiple-comparisons test was applied. *P ≤ 0.05, **P ≤ 0.01, ***P ≤ 0.001, and ****P < 0.0001.

The transcriptional role of AR has been widely considered both as a transcriptional activator, as well as a repressor [6]. To investigate whether AR directly participates in the transcriptional repression regulation of ARHGEF2, we examined the presence of putative AR binding sites (also known as androgen response elements, AREs) in the ARHGEF2 promoter region by using a transcription factor binding prediction website, JASPAR (the 8th release, http://jaspar.genereg.net). Further, through the CUT & Tag sequencing assay, we investigated the AR binding sites in LNCaP cells cultured with 10% CD-FBS and then stimulated with DHT for 24 h and identified the high-confidence peaks as potential AR binding sites in the ARHGEF2 loci (Supplementary Fig. 3). Based on an integrated analysis of CUT & Tag data and prediction data, two AREs within the ~2 kb region upstream of the transcription start site (TSS) of ARHGEF2 were selected (Fig. 3A). To confirm AR binding at the ARHGEF2 promoter sites, we performed ChIP-qPCR for AR in DHT-stimulated LNCaP and 22RV1 cells and used KLK3 as a positive control, and a significant enrichment for AR-binding at these two sites (ARE-1 and ARE-2) was observed upon DHT stimulation (Fig. 3B, C). To further confirm the AR-mediated transcriptional repression of ARHGEF2, we performed the luciferase reporter assay using proximal (ARHGEF2-PP) and distal (ARHGEF2-DP) promoter regions of ARHGEF2 in LNCaP and 22RV1 cells. Upon androgen stimulation, a concentration-dependent decrease in luciferase activity was observed in the LNCaP and 22RV1 cells transfected with ARHGEF2-PP and ARHGEF2-DP (Fig. 3D, E). A significant increase in the luciferase activity of both the reporter constructs was observed by 20uM enzalutamide treatment (Fig. 3F, G). Given the evidence that ARHGEF2 was robustly inhibited by DHT, we next examined whether silencing AR expression conferred any change in ARHGEF2 expression. Similar to the androgen inhibition, AR-specific siRNA exhibited robust increases in the mRNA expression of ARHGEF2 in LNCaP (~1.5-fold) and 22RV1 (~3-fold) cells (Fig. 3H–K). Furthermore, shRNA mediated knockdown of AR and AR splice-variant 7 (AR-V7) in LN95 cells (GSE106560) caused a 1.87-fold upregulation of ARHGEF2 expression by shAR compared to the control group, while no significant increase (~1.15-fold) was observed in shAR-V7 cells (Supplementary Fig. 4). These findings collectively indicate that AR acts as a transcriptional repressor of ARHGEF2.

### ARHGEF2 promotes neuroendocrine differentiation in PCa

**Fig. 4:**
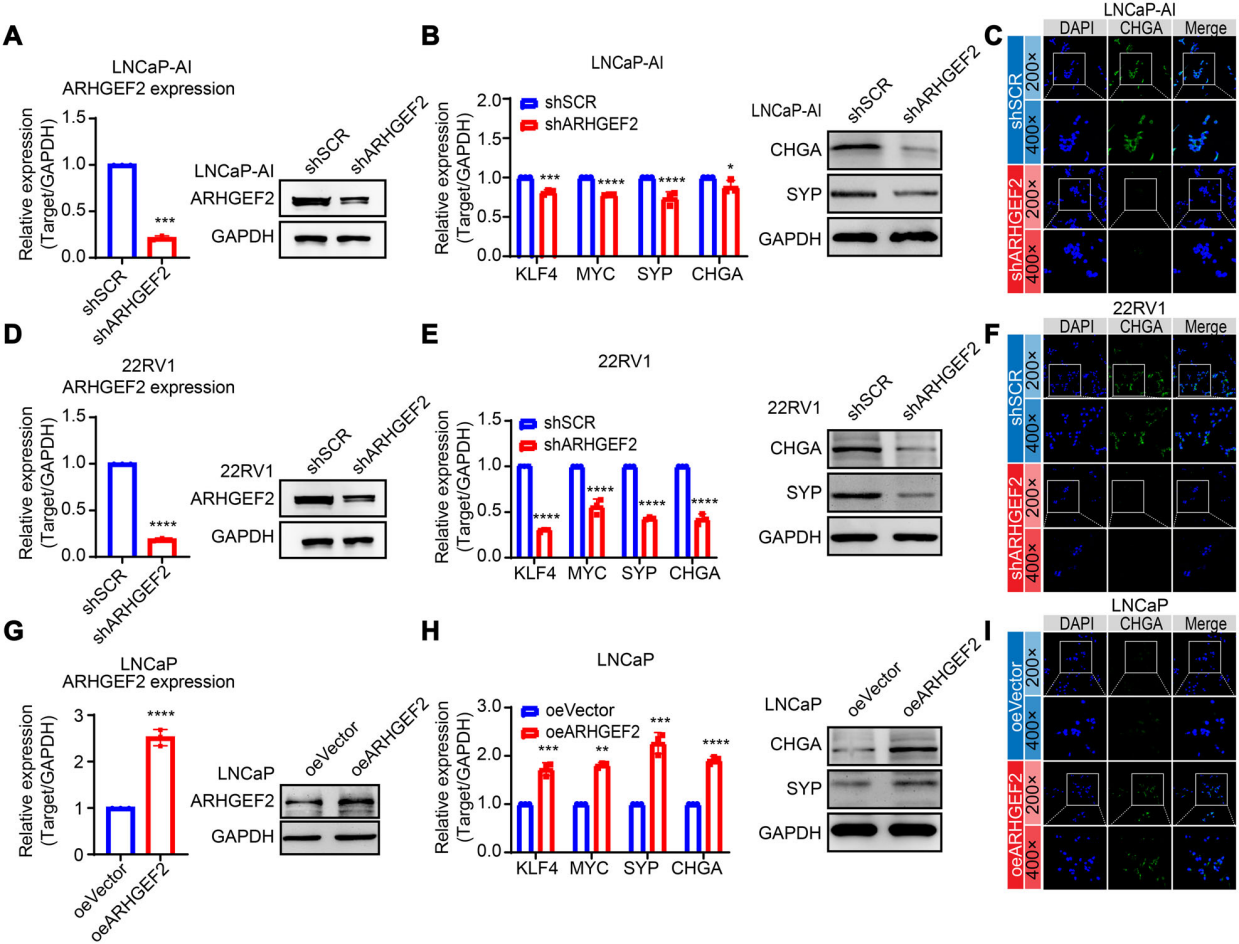
ARHGEF2 promotes the neuroendocrine differentiation in prostate cancer. **A** QPCR and Immunoblot data showing ARHGEF2 expression levels in ARHGEF2-silenced (shARHGEF2) and control (shSCR) LNCaP-AI cells. **B** QPCR data showing relative expression of KLF4, MYC, SYP and CHGA using same cells as **A**. Immunoblot data showing SYP and CHGA expression using same cells as **A**. **C** Immunostaining for CHGA in ARHGEF2-silenced (shARHGEF2) and control (shSCR) LNCaP-AI cells. **D** QPCR and Immunoblot data showing ARHGEF2 expression levels in ARHGEF2-silenced (shARHGEF2) and control (shSCR) 22RV1 cells. **E** QPCR data showing relative expression of KLF4, MYC, SYP and CHGA using same cells as **D**; Immunoblot data showing SYP and CHGA expression using same cells as **D**. **F** Immunostaining for CHGA in ARHGEF2-silenced (shARHGEF2) and control (shSCR) 22RV1 cells. **G** QPCR and Immunoblot data showing ARHGEF2 expression levels in ARHGEF2-overexpressed (oeARHGEF2) and control (oeVector) LNCaP cells. **H** QPCR data showing relative expression of KLF4, MYC, SYP and CHGA using same cells as **G**. Immunoblot data showing SYP and CHGA expression using same cells as **G**. **I** Immunostaining for CHGA using same cells as **G**. For panels **A**, **D**, **G** two-tailed unpaired Student’s t-test; **B**, **H**, **I** two-way ANOVA, Sidak’s multiple-comparisons test was applied. *P ≤ 0.05, **P ≤ 0.01, ***P ≤ 0.001, and ****P < 0.0001.

The ARHGEF2 pathway controls synaptic re-networking and overall gene expression through regulating cytoskeleton dynamics and may promote neuroplasticity [47]. To identify the role of ARHGEF2 in neuroendocrine differentiation, we established stable ARHGEF2-silenced LNCaP-AI cells (LNCaP-AI shARHGEF2) and scrambled control (LNCaP-AI shSCR) using lentivirus-based short-hairpin RNAs (Fig. 4A) and examined well-known NE markers. A mild reduction in KLF4, MYC, SYP, and CHGA (NE markers) expression was observed in LNCaP-AI shARHGEF2 cells compared to LNCaP-AI shSCR control cells (Fig. 4B, C), and likewise a more pronounced decrease was observed in 22RV1 shARHGEF2 cells of these NE markers (Fig. 4D–F), which highlights the role of ARHGEF2 in neuroendocrine differentiation. Conversely, ectopic overexpression of ARHGEF2 in LNCaP cells show a robust increase in the expression of NE markers (Fig. 4G-I). Taken together, these findings highlight the predominant role of ARHGEF2 in neuroendocrine differentiation in prostate cancer.

### ARHGEF2 regulate SOX2 via FGFR1/MAPK pathway in PCa

**Fig. 5:**
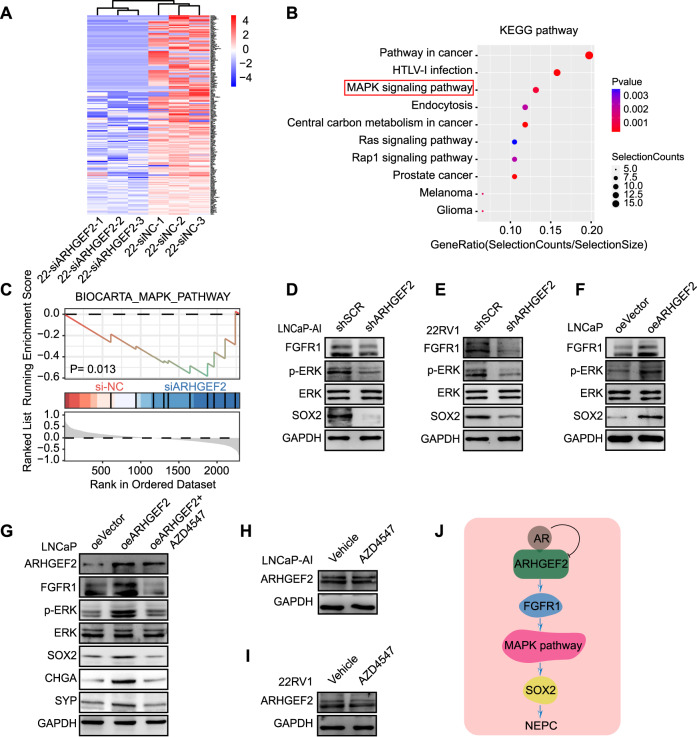
ARHGEF2 regulate SOX2 via FGFR1/MAPK pathway in PCa. **A** Heatmap showing genes up-regulated (red) or down-regulated (blue) in 22RV1 ARHGEF2 downregulated cells obtained by RNA-seq analysis. **B**, **C** KEGG pathway enrichment (**B**) and GSEA (**C**) analysis showing the MAPK pathway enriched in the 22RV1-siARHGEF2 group relative to control. **D**, **E** Immunoblot analysis for protein levels in SOX2 and FGFR1/MAPK pathway in ARHGEF2-silenced (shARHGEF2) and control (shSCR) LNCaP-AI (left) and 22RV1 (right) cells. **F** Immunoblot analysis for protein levels in SOX2 and FGFR1/MAPK pathway in ARHGEF2-overexpressed (oeARHGEF2) and control (oeVector) LNCaP cells. **G** Immunoblot analysis for protein levels in SOX2 and FGFR1/MAPK pathway using the same cells as **F** treated with AZD4547. AZD4547, a selective FGFR inhibitor [50]. **H**, **I** Immunoblot analysis for protein levels in ARHGEF2 using the LNCaP-AI and 22RV1 cells treated with Vehicle and AZD4547. **J** Illustration showing AR-repressed ARHGEF2 regulate SOX2 via FGFR1/MAPK pathway in prostate cancer. AR, androgen receptor; ARHGEF2, Rho guanine nucleotide exchange factor 2; FGFR1, fibroblast growth factor receptor 1; SOX2 SRY-Box transcription factor 2; NE neuroendocrine.

To identify the biological processes governed by ARHGEF2 and elucidate its functional relevance, RNA-seq and pathway analysis were performed. Two siRNAs were used to suppress the expression of ARHGEF2 in 22RV1 cells, which displayed the highest ARHGEF2 expression levels compared to LNCaP cells and performed RNA-seq analysis (Supplementary Fig. 5 and Fig. 5A). The knock-down effects were confirmed by western blot (Supplementary Fig. 6). Our analysis revealed 189 genes downregulated in 22RV1 siARHGEF2 cells (Fold Change cut-off: 2.0; P-value cut-off: 0.05), which were further analyzed for enriched KEGG pathways (Fig. 5B). Notably, genes downregulated by ARHGEF2 deletion were associated with critical pathways, namely, pathways in cancer, MAPK signaling pathway, central carbon metabolism in cancer and prostate cancer pathway. To address the MAPK signaling pathway enrichment, we performed Gene set enrichment analysis (GSEA). Compared to the negative control, the MAPK pathway showed lower enrichment scores upon ARHGEF2 inhibition (*p* = 0.013) (Fig. 5C). We next examined the expression of key modules (ERK phosphorylation) in the MAPK pathway and found a significant decrease in the shRNA-mediated ARHGEF2-silenced cells and a significant increase was also observed in the ARHGEF2 overexpressing LNCaP cells (Fig. 5D–F).

Gain of the Fibroblast Growth Factor Receptor 1 (FGFR1) is reported associated with the progression to malignancy in PCa and many other epithelial originating lesions [48]. Our RNA-seq data show reduced FGFR1expression upon ARHGEF2 deletion, we sought to examine ARHGEF2-mediated regulation of FGFR1 and FGFR1/MAPK signaling pathway. We examined the FGFR1expression in two ARHGEF2 deletion cell lines, and ectopic overexpression of ARHGEF2 LNCaP cells (Fig. 5D–F). Using these cells, we observed a remarkable decrease of FGFR1 at ARHGEF2 deletion cell lines (Fig. 5D, E). Similarly, in ectopic overexpression of ARHGEF2 LNCaP cells, FGFR1 also exhibit a significant upregulation along with ARHGEF2 overexpression (Fig. 5F). Besides, FGFR1 activation could upregulate SOX2 expression by downstream phosphorylated ERK of MAPK pathway [49]. Our data also show reduced ARHGEF2 expression could downregulate FGFR1 and SOX2 expression (Fig. 5D, E), we sought to examine FGFR1-mediated regulation of SOX2. The upregulation of SOX2 by ARHGEF2 overexpression was suppressed by FGFR1 inhibitor AZD4547 [50] (Fig. 5G). As expected, the FGFR1 inhibitor AZD4547 treatment showed no impact on ARHGEF2 expression (Fig. 5H, I). These data confirmed that ARHGEF2 function via FGFR1/MAPK pathway to regulate SOX2 (Fig. 5J).

### ARHGEF2 is important for prostate cancer cell growth

**Fig. 6:**
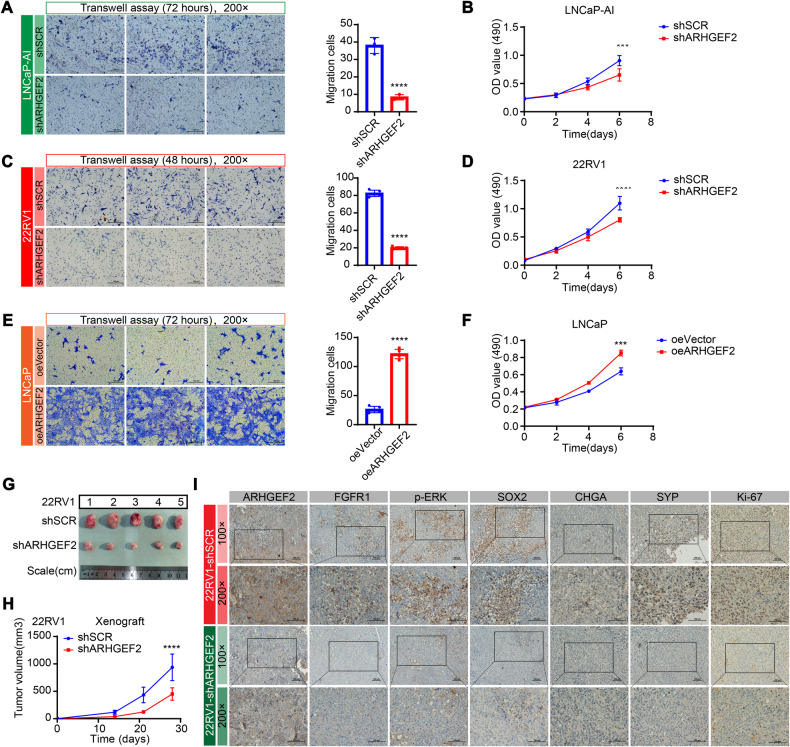
Targeting ARHGEF2 reduces the tumor growth of prostate cancer cells. **A** Transwell migration assay in LNCaP-AI cells infected with lentiviruses carrying shARHGEF2. The left panel shows the representative microphotographs from a single independent experiment (scale bar = 100 µm). **B** MTT assays in LNCaP-AI cells infected with lentiviruses carrying shARHGEF2. Cell growth assessed daily for 6 days using an MTT assay in LNCaP-AI cells. Data were obtained from three independent experiments with samples in triplicate. **C** Transwell migration assay in 22RV1 cells infected with lentiviruses carrying shARHGEF2. The left panel shows the representative microphotographs from a single independent experiment (scale bar = 100 µm). **D** MTT assays in 22RV1 cells infected with lentiviruses carrying shARHGEF2. Cell growth assessed daily for 6 days using an MTT assay in 22RV1 cells. Data were obtained from three independent experiments with samples in triplicate. **E**, **F** Transwell migration assay (**E**) from a single independent experiment and MTT assays (**F**) in LNCaP cells infected with lentiviruses carrying overexpressed ARHGEF2 (oeARHGEF2). **G** Representative image of the dissected tumors was shown. **H** Growth curves of xenografts of 22RV1 cells infected with shSCR or shARHGEF2. Data are representative of mean ± SD of *n* = 5 tumors per group. **I** Representative image of the dissected tumors was shown. Representative images showing immunostaining (×100 and ×200 magnification) for ARHGEF2, FGFR1, p-ERK, SOX2, CHGA, SYN and Ki-67 in tumor specimens obtained from xenografts. For panels **A**, **C**, **E** two-tailed unpaired Student’s t-test; For panels **B**, **D**, **F**, and **H**, two-way ANOVA, Sidak’s multiple-comparisons test was applied. *P ≤ 0.05, **P ≤ 0.01, ***P ≤ 0.001, and ****P < 0.0001.

To examine the biological functions governed by ARHGEF2, stable ARHGEF2-silencing cell lines were used as described above (Fig. 4A, D). ARHGEF2 knockdown significantly reduced cell migration in LNCaP-AI and 22RV1 cells (Fig. 6A, C). ARHGEF2 suppression also suggested an apparent effect on cell proliferation relative to control (shSCR group) in LNCaP-AI and 22RV1 cells (Fig. 6B, D). Conversely, ARHGEF2 overexpression significantly promoted cell migration and proliferation in LNCaP cells (Fig. 6E, F).To further evaluate the cellular functions of ARHGEF2 in CRPC cells, flow cytometry was performed to assess the cell cycle. The cell cycle was arrested in the S phase after ARHGEF2 was silenced (Supplementary Fig. 7). In addition to the in vitro data, we examined the contribution of ARHGEF2 to tumor growth in vivo (Fig. 6 G–I). The 22RV1 cells with the ARHGEF2 deletion exhibited profound attenuation of tumor growth relative to the control group (Fig. 6H). Also, a remarkable decrease in the ARHGEF2 expression accompanied by FGFR1/MAPK pathway and NE markers (CHGA and SYP) was observed by IHC in tumors of the ARHGEF2 knockdown mice, thus reaffirming the association between ARHGEF2 and NE-like phenotype (Fig. 6I). These data support the model that ARHGEF2 is essential to the growth of PCa cells.

## Discussion

AR functions as a transcriptional activator and is involved in the progression of prostate cancer with prostate-specific antigen (PSA) being a well-established AR-induced gene. As far as current treatment is concerned, targeting androgen signals through surgery or chemical castration remains as the mainstay treatment for malignant PCa. However, despite an initial regression and initial survival benefit observed, the disease inevitably comes back in a more aggressive state, called CRPC [51]. Mechanisms, such as AR amplification, AR alternative splicing, AR crossover activation, or AR bypass pathway, may transform to a resistance status in patients to AR-targeted therapies [12]. Studies have also shown that AR-targeted therapies de-repress both onco-suppressor genes and oncogenes that are normally inhibited by AR/AR signaling [11].

Recent studies have underlined the oncogenic role of ARHGEF2 in promoting breast cancer cell invasion and metastasis and in the brain metastatic behavior of melanoma cells [52, 53]. However, the mechanism by which ARHGEF2 is regulated in PCa and why its up-regulation is associated with an aggressive phenotype remains unclear. Here, we provide sufficient evidence that ARHGEF2 is an androgen-suppressing gene, and the use of AR antagonist or ADT treatment can relieve the AR signal-mediated transcriptional inhibition of ARHGEF2, leading to the upregulation of ARHGEF2. Combined with our experiments, the expression of ARHGEF2 and AR was negatively correlated during immunohistochemical staining of PCa specimens, proving that AR/AR signaling regulates ARHGEF2.

Studies have pointed out that the ARHGEF2-mediated feedback loop increases the flux control of other pathways through the MAPK pathway [54–56]. The overexpression of ARHGEF2 is sufficient to increase ERK1/2 phosphorylation raising the possibility that the oncogenic potential of ARHGEF2 is partially mediated through its capacity to activate the MAPK pathway in pancreatic cancer. Our data suggest that ADT-induced ARHGEF2 is involved in controlling the flux of the MAPK pathway via FGFR1, which is consistent with the above-mentioned model. It is plausible that by ARHGEF2 the coordinated activation of specific proteins in the MAPK pathway, may predispose malignant cancer cells to morphological alterations and the acquired invasive behavior that promotes tumor cell dissemination, which are key steps leading to acquiring resistance and driving the NE phenotype. Additionally, it’s been reported that the role of SRY-box 2 (SOX2) has been implicated in promoting lineage plasticity and antiandrogen resistance in TP53- and RB1-deficient PCa [57].

Here, we demonstrate that SOX2 is regulated by ARHGEF2 via the FGFR1-induced MAPK pathway in PCa. Although our initial results revealed the important role of ARHGEF2 in PCa for the first time here, this is still only a preliminary study. Firstly, given the finding that ARHGEF2 amplification occurs in ~30% CRPC patients, which in a way may explain the upregulation was observed in CRPC patients when compared with primary PCa patients; however, the mechanisms behind this genetic change (ARHGEF2 amplification) still need to be further studied. Secondly, as Fig. 1A showed, the ARHGEF2 expression level differs between benign prostate tissues and primary PCa samples. The ARHGEF2 expression level was decreased when compared to primary PCa patients, especially those with GS < 7. We speculated that this may be due to the different AR activity—as PSA levels rise in most of the primary PCa when first diagnosed—this relatively active AR activity would repress ARHGEF2 expression at the transcription level. This in turn led to a reduction in the expression level of ARHGEF2 in primary PCa. More evidence to further elucidate the related mechanisms in order to better explain this phenomenon are needed. Moreover, AZD4547, a novel selective small-molecule inhibitor of FGFR, showed potent antitumor activity in FGFR-dependent tumors [50]. To this end, we observed that AZD4547 treatment did inhibit the growth of PCa cell lines (Supplementary Fig. 8). AZD4547 in combination with enzalutamide treatment for advanced PCa patients is conceivable, but further investigation is again needed. Lastly, further research, i.e., overexpressing ARHGEF2 in CRPC or enzalutamide-resistant models and using patient-derived organoids which harbor AR deletion, low AR activity or no AR expression models may be a more interesting way to demonstrate ARHGEF2 function in prostate cancer.

In summary, our findings emphasize that AR-targeted therapy for PCa patients may lead to increased levels of ARHGEF2. Although androgen ablation therapy is an effective treatment for PCa patients, it will ultimately develop resistance. Moreover, we show evidence that ARHGEF2 is highly expressed in patients with NEPC, which drives the NE lineage phenotype. Understanding the mechanism underlying NEPC progression and treatment resistance will eventually lead to more effective treatment strategies and improve the prognosis of patients who have already exhausted all currently available treatment measures.

### Supplementary information

checklist

Supplementary information

Original Data File

## Data Availability

The gene expression data for DHT-treated LNCaP cells generated in this study has been submitted to the NCBI Gene Expression Omnibus under the accession number GSE163539. The gene expression data and binding data in this study have been deposited into CNGB Sequence Archive (CNSA) of China National GeneBank DataBase (CNGBdb) with accession numbers CNP0001560 and CNP0001628. There are various other datasets used in the study, namely: ChIP-seq dataset for AR binding without androgen stimulation in LNCaP cells, GSE125245, RNA-seq dataset of VCaP and 22RV1 cells with and without androgen (R1881) stimulation, GSE71797, RNA-seq dataset of shAR-V7 and shAR-FL mediated knock-down experiments in LN95 cells, GSE106560, RNA-seq dataset of both CRPC-Adeno and CRPC-NE samples (Beltran Cohort), dbGap phs000909.v.p1. The databases used in this study include: cBioPortal (http://www.cbioportal.org/) and Prostate Cancer Transcriptome Atlas (PCTA) website (http://www.thepcta.org/).
