# Correction: Androgen deprivation restores ARHGEF2 to promote neuroendocrine differentiation of prostate cancer

**DOI:** 10.1038/s41419-023-06051-0

**Published:** 2023-10-02

**Authors:** Xuanrong Chen, Yi Shao, Wanqing Wei, Shimiao Zhu, Yang Li, Yutong Chen, Hanling Li, Hao Tian, Guijiang Sun, Yuanjie Niu, Zhiqun Shang

**Affiliations:** 1 Department of Urology, Tianjin Institute of Urology, The second hospital of Tianjin Medical University, Tianjin, China; 2 Department of Pediatric Surgery, Huai’an Maternal and Children Health Hospital, Huai’an, China

**Keywords:** Prostate cancer, Mechanisms of disease

Correction to: *Cell Death and Disease* 10.1038/s41419-022-05366-8, published online 05 November 2022

In this article the caption of Fig. 6 has been given erroneously. It should be read:

Fig. 6. **Targeting ARHGEF2 reduces the tumor growth of prostate cancer cells**. **A** Transwell migration assay in LNCaP-AI cells infected with lentiviruses carrying shARHGEF2. The left panel shows the representative microphotographs from a single independent experiment (scale bar = 100 µm). **B** MTT assays in LNCaP-AI cells infected with lentiviruses carrying shARHGEF2. Cell growth assessed daily for 6 days using an MTT assay in LNCaP-AI cells. Data were obtained from three independent experiments with samples in triplicate. **C** Transwell migration assay in 22RV1 cells infected with lentiviruses carrying shARHGEF2. The left panel shows the representative microphotographs from a single independent experiment (scale bar = 100 µm). **D** MTT assays in 22RV1 cells infected with lentiviruses carrying shARHGEF2. Cell growth assessed daily for 6 days using an MTT assay in 22RV1 cells. Data were obtained from three independent experiments with samples in triplicate. **E**, **F** Transwell migration assay (**E**) from a single independent experiment and MTT assays (**F**) in LNCaP cells infected with lentiviruses carrying overexpressed ARHGEF2 (oeARHGEF2) **G** Representative image of the dissected tumors was shown. **H** Growth curves of xenografts of 22RV1 cells infected with shSCR or shARHGEF2. Data are representative of mean ± SD of *n* = 5 tumors per group. **I** Representative image of the dissected tumors was shown. Representative images showing immunostaining (×100 and ×200 magnification) for ARHGEF2, FGFR1, p-ERK, SOX2, CHGA, SYN and Ki-67 in tumor specimens obtained from xenografts. For panels **A**, **C**, **E** two-tailed unpaired Student’s t-test; For panels **B**, **D**, **F**, and **H**, two-way ANOVA, Sidak’s multiple-comparisons test was applied. *P ≤ 0.05, **P ≤ 0.01, ***P ≤ 0.001, and ****P ≤ 0.0001.

The original article has been corrected.

