# Supplementary material for: Androgen deprivation restores ARHGEF2 to promote neuroendocrine differentiation of prostate cancer
Source: Cell Death Dis. 2022 Nov 5;13(11):927. doi: 10.1038/s41419-022-05366-8 (PMC9637107; doi:10.1038/s41419-022-05366-8)
Supplement: Supplementary file 2 — Supplementary information [file 41419_2022_5366_MOESM2_ESM.docx]

**Supplementary information**

**Androgen deprivation restores ARHGEF2 to promote neuroendocrine differentiation of prostate cancer**

Xuanrong Chen^1,^ *, Yi Shao^1,^ *, Wanqing Wei^1, 2^ ^,^ *, Shimiao Zhu^1,^ *, Yang Li^1^, Yutong Chen^1^, Hanling Li^1^, Hao Tian^1^,Guijiang Sun^1^, Yuanjie Niu^1^, Zhiqun Shang^1, #^

*These authors contributed equally to this work.

**Authors Affiliations:**^1^Department of Urology, Tianjin Institute of Urology, The second hospital of Tianjin Medical University, Tianjin, 300211, China. ^2^Department of Pediatric Surgery, Huai'an Maternal and Children Health Hospital, Huai'an, 223002, China.

**^#^Corresponding Author:** Tianjin Institute of Urology, the Second Hospital of Tianjin Medical University, Pingjiang Rd 23#, Hexi District, Tianjin, 300211, China. E-mail address: zhiqun_shang@tmu.edu.cn (Zhiqun Shang).

Supplementary Figures 1-8

Supplementary Tables 1-4


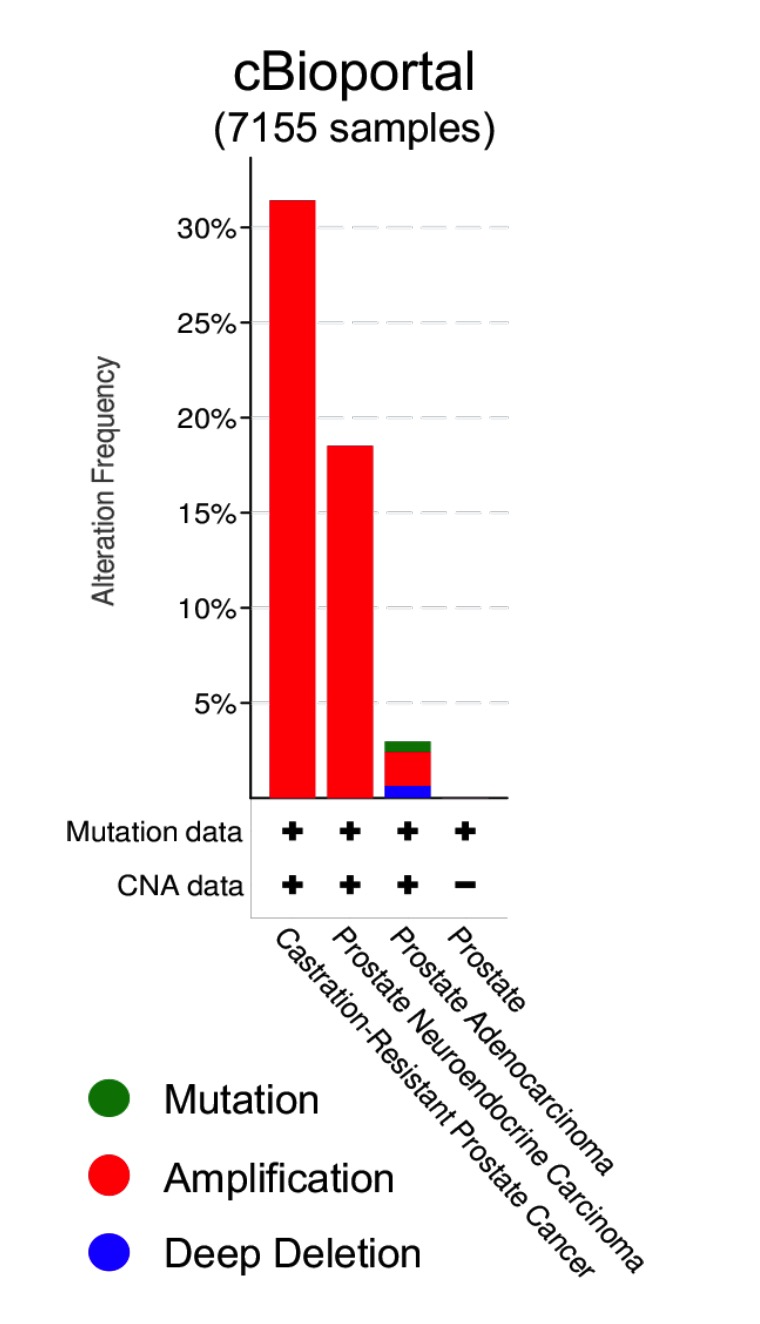


***Supplementary Figure 1. The genetic alteration pattern of ARHGEF2 in human prostate cancer samples.***

The genetic alteration pattern of ARHGEF2 in human prostate cancer samples from the cBioportal website (http://www.cbioportal.org). The data showed that over 30% CRPC and over 15% NEPC patients harbored ARHGEF2 gene amplification status.


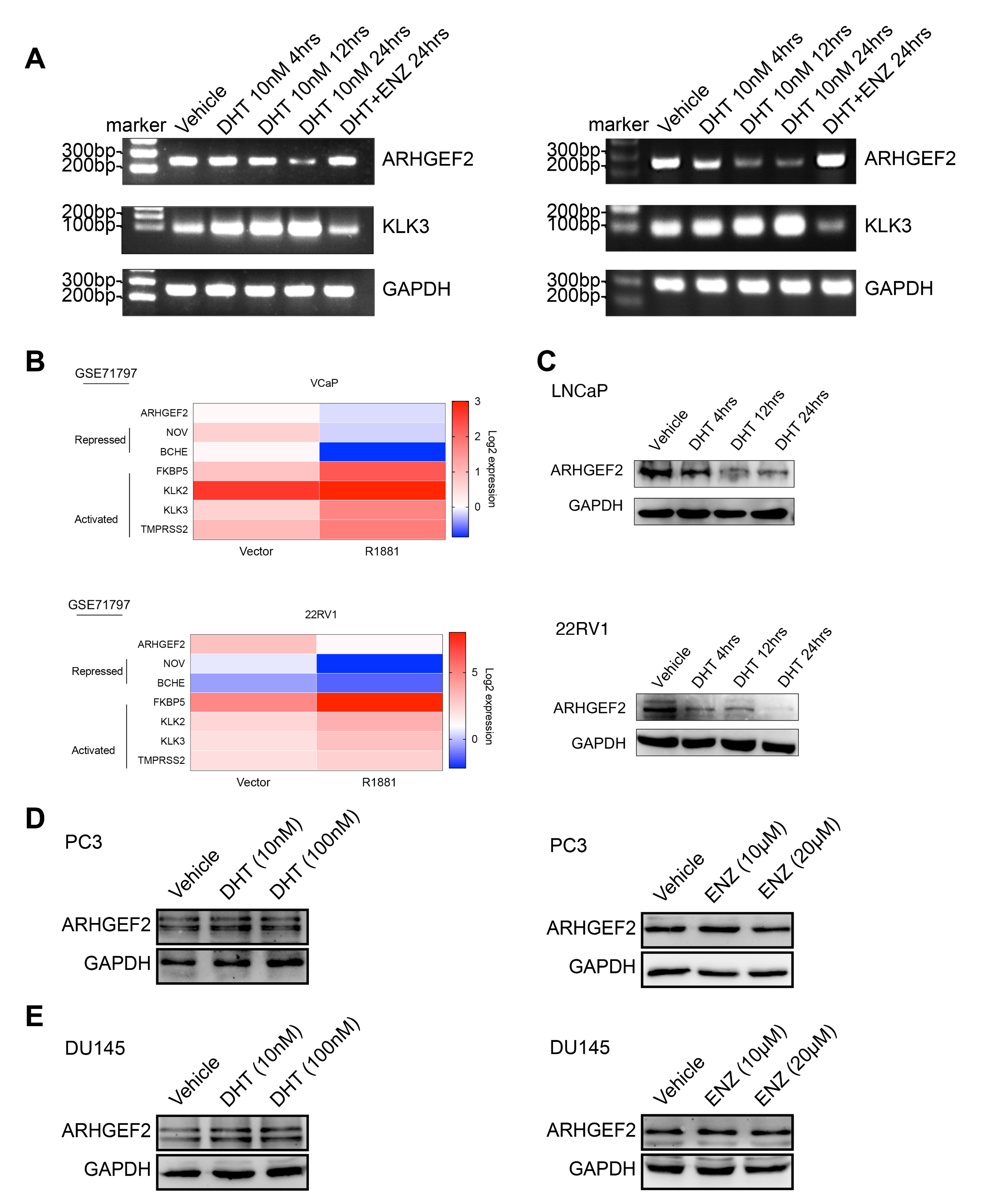


***Supplementary Figure 2. Gene expression profile of DHT-treated LNCaP cells.***

(A) End-point RT-PCR analysis of ARHGEF2 and KLK3 mRNA during various DHT (10nM) treatment periods on LNCaP (left) and 22RV1 (right) cell lines.

(B) Heatmap depicting relative expression of androgen regulated genes in androgen stimulated VCaP (GSE71797) and 22RV1 cells (GSE71797).

(C) Immunoblot for ARHGEF2 in LNCaP and 22RV1 cells stimulated for several DHT (10nM) treatment periods.

(D) Immunoblot for ARHGEF2 and GAPDH in PC3 cells stimulated with DHT (left) and enzalutamide treatment (right).

(E) Immunoblot for ARHGEF2 and GAPDH in DU145 cells stimulated with DHT (left) and enzalutamide treatment (right).

***Supplementary Figure 3. General information of CUT-Tag sequencing assay in LNCaP cells cultured with 10% CD-FBS followed by stimulation with DHT 24 hr.***

Cut-Tag (ChIP-seq) track of AR overlapping signals (red: AR binding status in DHT treatment) at KLK3 gene region as a positive control to evaluate the confidence of this assay.


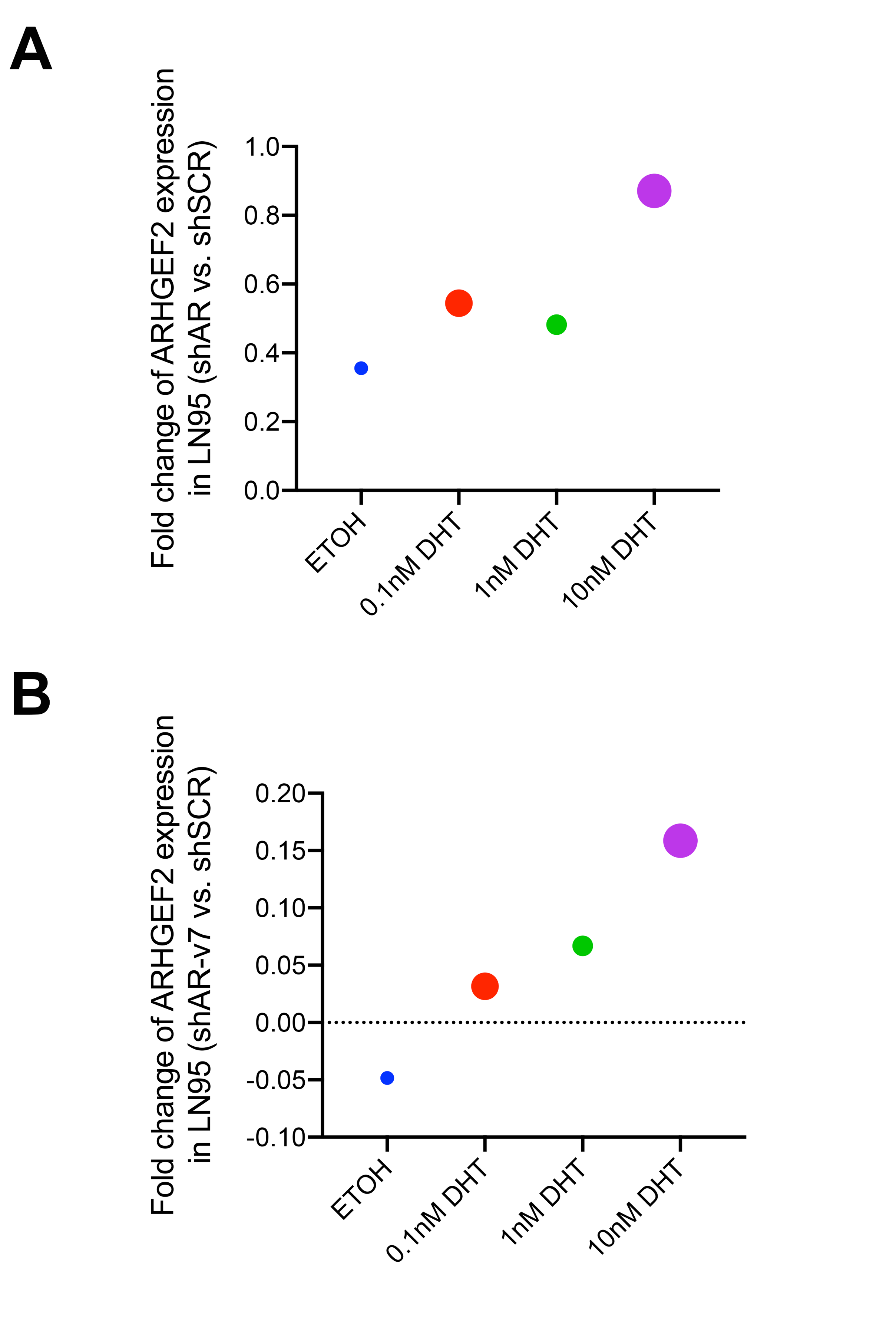


***Supplementary Figure 4. Bioinformatic analysis of RNA-seq data from shAR-V7 and shAR-FL mediated knock-down experiments in LN95 cell lines.***

The RNA-seq data was retrieved from GSE106560. (A) Log2 Fold change of ARHGEF2 expression in response to AR-FL knocked down (Y axis) and DHT stimulation (X axis) was presented. (B) Log2 Fold change of ARHGEF2 expression in response to AR-V7 knocked down (Y axis) and DHT stimulation (X axis) was presented. DHT, double hydrogen testosterone.


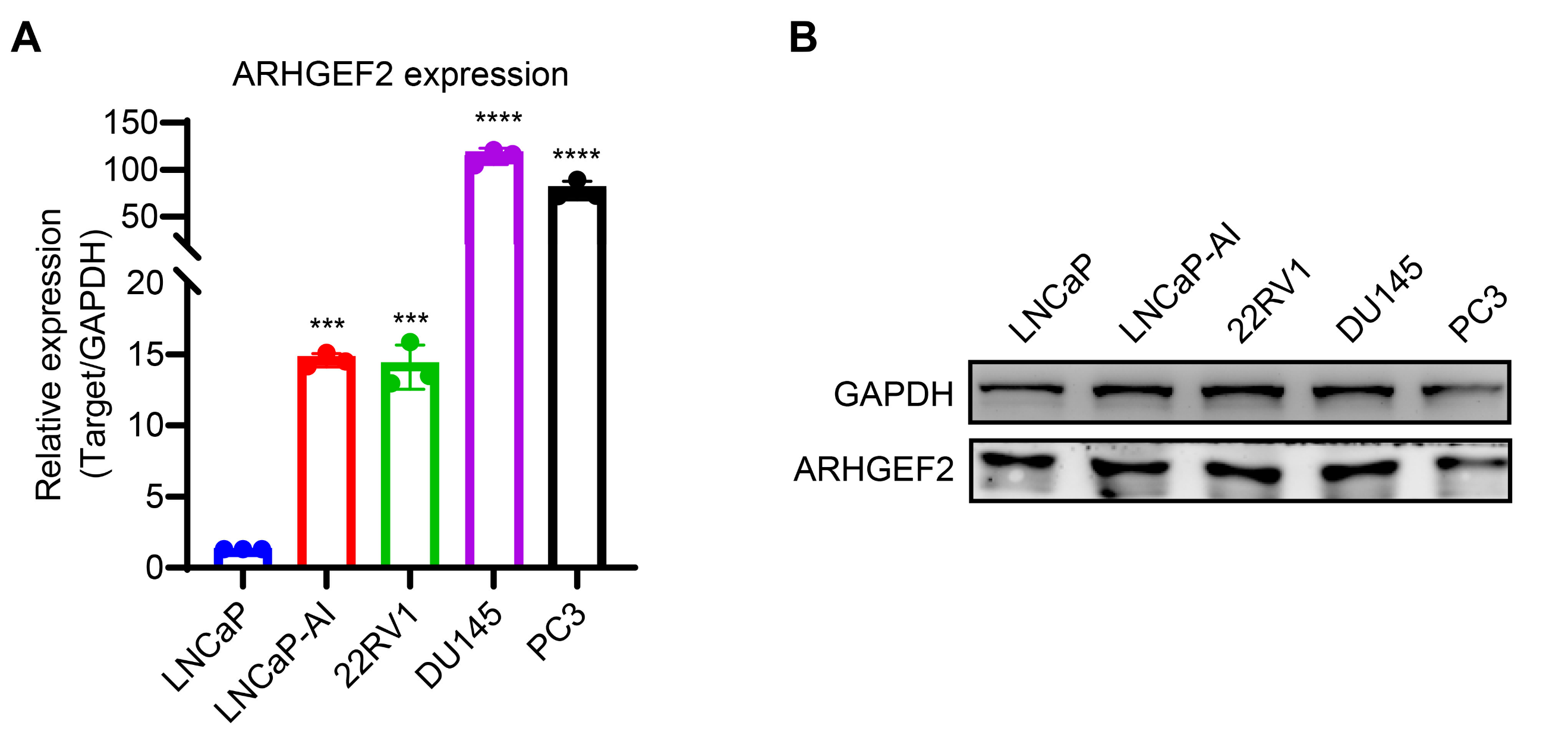


***Supplementary Figure 5. ARHGEF2 expression pattern in prostate cancer cells.***

QPCR data showed relative expression of ARHGEF2 in prostate cancer cell lines. 22RV1 cells displayed the highest ARHGEF2 expression levels compared to LNCaP cells.

**
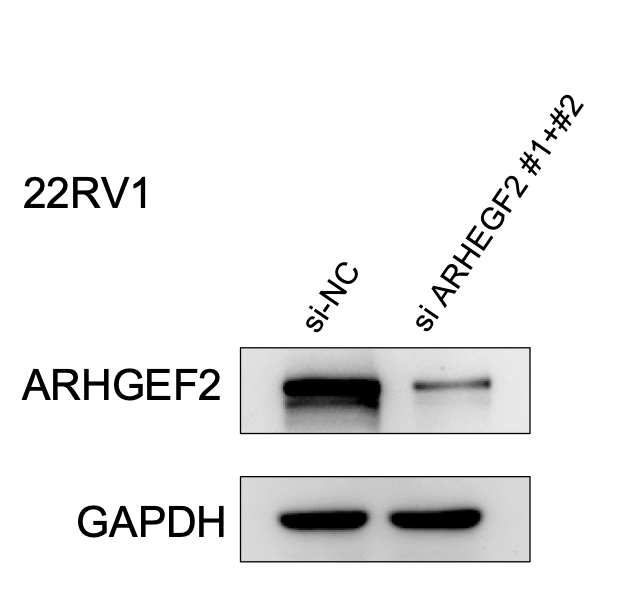
**

***Supplementary Figure 6. Inhibition of ARHGEF2 in PCa cells.***

Immunoblot analysis for ARHGEF2 levels in ARHGEF2-silenced (siARHGEF2 #1 + #2) and control (si-NC) 22RV1 cells (These cells used for RNA-seq analysis).

***
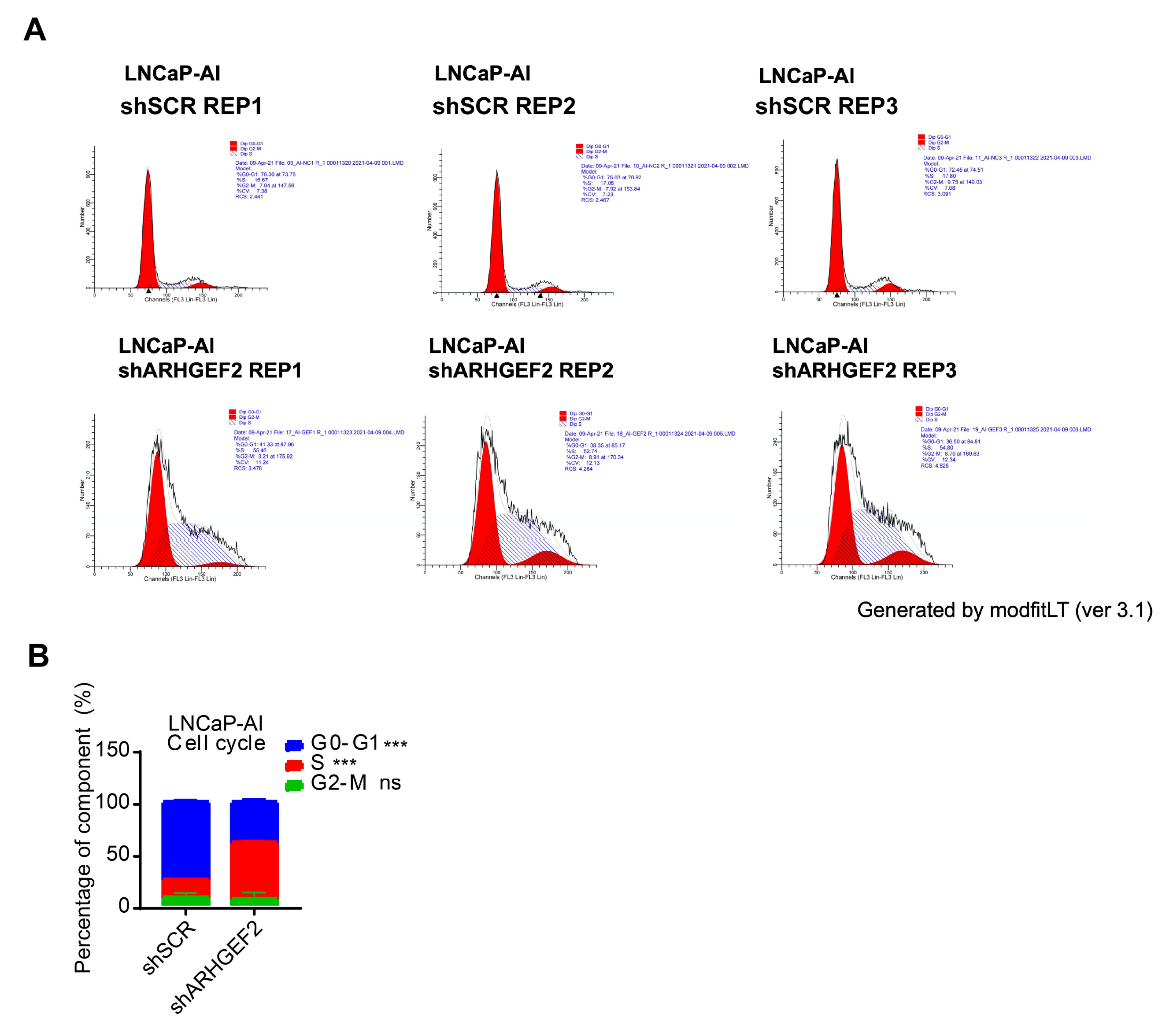
***

***Supplementary Figure 7. ARHGEF2 regulates cell cycle in PCa cells.***

Cell cycle analysis in ARHGEF2-silenced (shARHGEF2) and control (shSCR) LNCaP-AI cells.

**
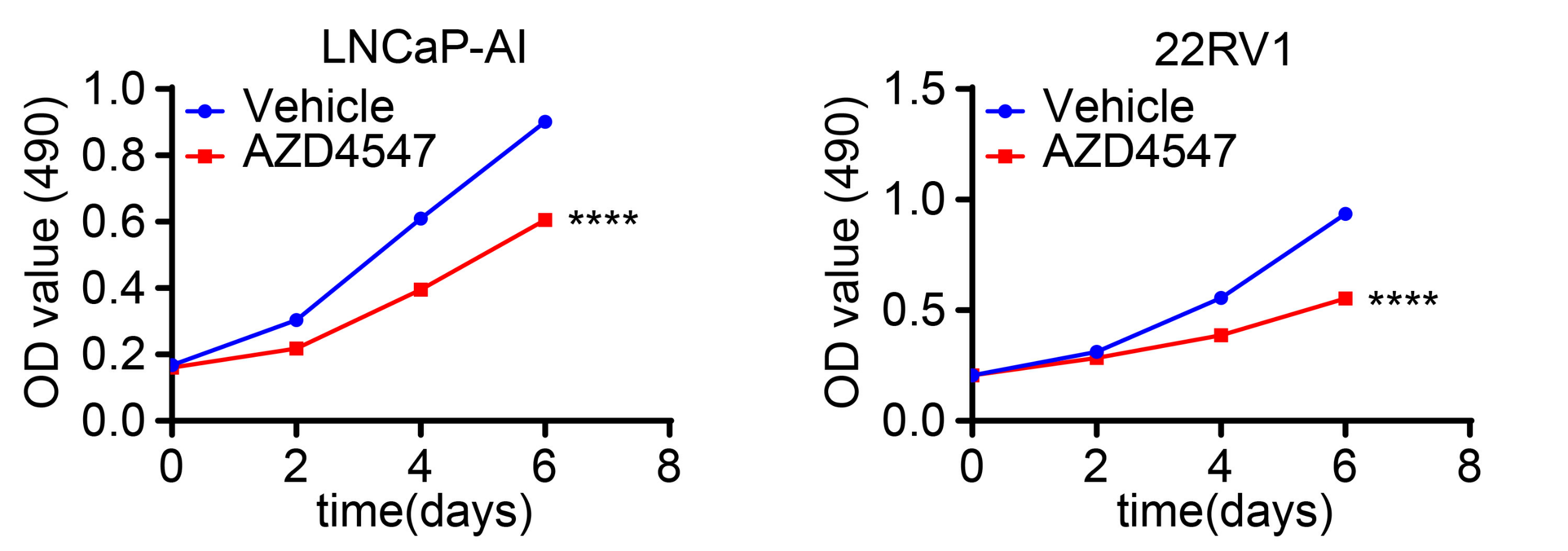
**

***Supplementary Figure 8. AZD4547 treatment in LNCaP-AI and 22Rv1 cells.***

MTT assays in LNCaP-AI and 22RV1 cells treated with Vehicle and AZD4547. Cell growth assessed daily for 6 days using an MTT. Data were obtained from three independent experiments with samples in triplicate.

***Supplementary Table 1. The siRNA sequences used for silencing ARHGEF2 and AR.***

| siRNA | Forward (5’-3’) | Reverse (5’-3’) |
| --- | --- | --- |
| siARHGEF2 #1***** | CCAAGUACCCGUUACUCAUTT | AUGAGUAACGGGUACUUGGTG |
| siARHGEF2 #2 | GCUUACCUGCGGCGAAUUATT | UAAUUCGCCGCAGGUAAGCCT |
| siAR #1 | GACUCAGCUGCCCCAUCCATT | UGGAUGGGGCAGCUGAGUCTT |
| siAR #2 | CCGGAAGCUGAAGAAACUUGGUAAU | AUUACCAAGUUUCUUCAGCUUCCGG |
| Scramble***** | UUCUUCGAACGUGUCACGUTT | ACGUGACACGUUCGGAGAATT |

(*, Used for generating shRNA)

***Supplementary Table 2. Primers used for gene expression analysis.***

| Primer | Forward (5’-3’) | Reverse (5’-3’) |
| --- | --- | --- |
| GAPDH | CGGAGTCAACGGATTTGGTC | TCGCCCCACTTGATTTTGGA |
| ARHGEF2 | CCCTCATTGACGAAGCAGAG | GTAGCTCTTCCAGCATCCCC |
| AR | CAGCCTATTGCGAGAGAGCTG | GAAAGGATCTTGGGCACTTGC |
| KLK3 | CAGTCTGCGGCGGTGTT | GCAAGATCACGCTTTTGTTCCT |
| KLF4 | CCCACATGAAGCGACTTCCC | CAGGTCCAGGAGATCGTTGAA |
| MYC | GGCTCCTGGCAAAAGGTCA | CTGCGTAGTTGTGCTGATGT |
| SYP | GCAATGGGTCTTCGCCATCT | GCCTGAAGGGGTACTCGAAC |
| CHGA | ACTGCGCTCCCTGTGAAC | GGGCTTGGAAAGTGTGTCGG |

***Supplementary Table 3. Primers used for ChIP-qPCR analysis.***

| Primer | Forward (5’-3’) | Reverse (5’-3’) |
| --- | --- | --- |
| ARE-1 | CTAGGACCCTCGTACCTCTC | CCAGCACCAGTTCCTCAAA |
| ARE-2 | CGTAGGTATACTTGGCAGGTG | TTCAGGCTGGATGGACATTAG |
| KLK3 | CCTAGATGAAGTCTCCATGAGCTACA | GGGAGGGAGAGCTAGCACTTG |

***Supplementary Table 4. The DNA sequences for luciferase analysis.***

| Plasmid | pGL3-ARHGEF2 PP (proximal promoter) | pGL3-ARHGEF2 DP (Distal promoter) |
| --- | --- | --- |
| Restriction Enzyme cutting (5’) | NheI | NheI |
| Restriction Enzyme cutting (3’) | XhoI | XhoI |
| Insert DNA sequences | AGGCCAGGAAGGAACTGGTCCTGGGTTTTCTAAGAGCTGGGGGCGGATGGAATTCTCCTGCCTGCGGGTCTTTAAAGACTGAGGTAACGCAGTAGGGGAGGGAAAAAGGGAGGGCGAGGGGAGAAGGGAAGAAGGGGAGGGCAGAGGGGAAAGGGCCAACCTGGGTCCGGCGAGGGCTGCGGGGCTGGGTTTTAGGAAAAGAAGAGCTAAGAGGCGGGTCTCTGGGGCTAGAGGGCGCCCCCAGGCGCTGGAGGGGAAATAGGCAGAGGGGCCTCTCAAACCTGTCCTAGGACCCTCGTACCTCTCCGAGCTGCCCCCACACAAACCCTGGCGTCCCGTCCTGCGCGCGGGCACCCCGCCCTCCCTCCGCTCCGCCTACCCCCTTCCTGAGCTTGGGCGGGGGAGGAGACTCGGGTTTGAGGAACTGGTGCTGGGTGCCGGGCGAGGGGAGTCAGACTTCCTGTCCCCGAGACCAACGCGTGCGGGCCGAACCCCTCCCC | CAGGAGCCTCAGGCCTCTTGGTTTGGGGGAGTTCCTACACCCTCCCCGTAGGTATACTTGGCAGGTGCAAGCCAGGAAGAACAAGCTGTGGCCCTGGGGAAGTCCTTCCTGCTTTCTAATGTCCATCCAGCCTGAACTGGGCACGGTTTATTTCCTCTTCCTTTTTCCTGGTCTTTGCTATTTAAGGATTCTAAACAGGGGAATGGGTGAAGAAGGAGGTGAAGGAGTTGCTAGGAAGGAGGGGAAGCCGGAGGGGCAGTGTGGGGGTAGTGCTGGGGGTGAGGAGCCCAACCAGCTCAGCCAGTGGTGAGGTAGTGGAGCCCAAAAATAGTGGCAGAGGCTAAAATTGGCACCTACAAGAGTGCTGAGTTGGGCTTGAAACCTGCAGCCTGGCCCCACTGCTGAGCCAGGGCAGAGAACTGCTGGATCTGGGTTGGGTCTCCCCTCCCTCCCTTCTTCTCCCACGCCCTTTGGGGCCAGAATCAGGCAGGACCCTGCTAACCGAAGGCTCTGTCCCAGGCCTCTCTGTGCTTCTGGTTTCTCCAGAGAAATGGGAGACCCTGAGAGGCAGAAATGAACTTCACTGTAAGTGATCATAGTTAGACTGGAGGAGGAATTTTCCCAATAGCAGGAAACTCACCCAAAGGCTGGCTCAGGCTGGGGAAAAGGGTCCAGAGAGATGAATGCCCCACCTTACCCTGCTTCTTGGGGAGAGGCTGGTTGGGCCCTAACTTTCTTTGACTAGGGCAGGCTGGTTTCTGGTCTTTCAGTTCCGGCTTAACTTGCAACAGAGAGAACTGCAGTTAGACACATGGAAGGCCTTCGGTAAAAGTGGAGAATCCCAACCTAGTCCCCCAAAGTAGCTGGCAGAGGCGGAGCTAGGAGGAGGGCAGAGTGGGGTCAGGAAGGCCCCAGAAGGGTGGGGCATGTGGGAGAATTCTCTCCTCTCTGTTCTTCGACCTCTGGGAGTGAATCCTACCCTTCCCGTGTACTGAAGATCCAGCGTTTAGTTCTCCTTTGATTTCCTCTCCCTCTGGCCACCCCTGCCCCCAATCGTAAAAGGGGTCAGTCTGCTCAGGCCTGCTTTGATGGGACCCCGAAGGCCAGGAAGGAACTGGTCCTGGGTTTTCTAAGAGCTGGGGGCGGATGGAATTCTCCTGCCTGCGGGTCTTTAAAGACTGAGGTAACGCAGTAGGGGAGGGAAAAAGGGAGGGCGAGGGGAGAAGGGAAGAAGGGGAGGGCAGAGGGGAAAGGGCCAACCTGGGTCCGGCGAGGGCTGCGGGGCTGGGTTTTAGGAAAAGAAGAGCTAAGAGGCGGGTCTCTGGGGCTAGAGGGCGCCCCCAGGCGCTGGAGGGGAAATAGGCAGAGGGGCCTCTCAAACCTGTCCTAGGACCCTCGTACCTCTCCGAGCTGCCCCCACACAAACCCTGGCGTCCCGTCCTGCGCGCGGGCACCCCGCCCTCCCTCCGCTCCGCCTACCCCCTTCCTGAGCTTGGGCGGGGGAGGAGACTCGGGTTTGAGGAACTGGTGCTGGGTGCCGGGCGAGGGGAGTCAGACTTCCTGTCCCCGAGACCAACGCGTGCGGGCCGAACCCCTCCCC |
