# Supplementary material for: Androgen deprivation restores ARHGEF2 to promote neuroendocrine differentiation of prostate cancer
Source: Cell Death Dis. 2022 Nov 5;13(11):927. doi: 10.1038/s41419-022-05366-8 (PMC9637107; doi:10.1038/s41419-022-05366-8)

Figure 3E

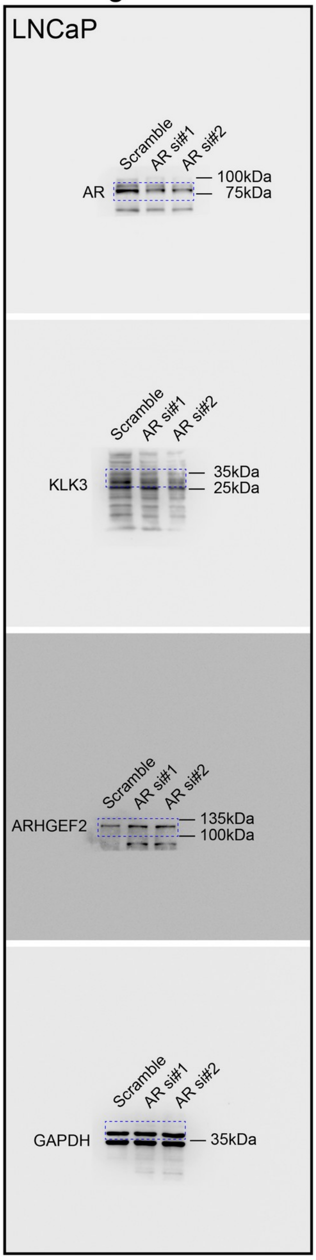

Figure 3F

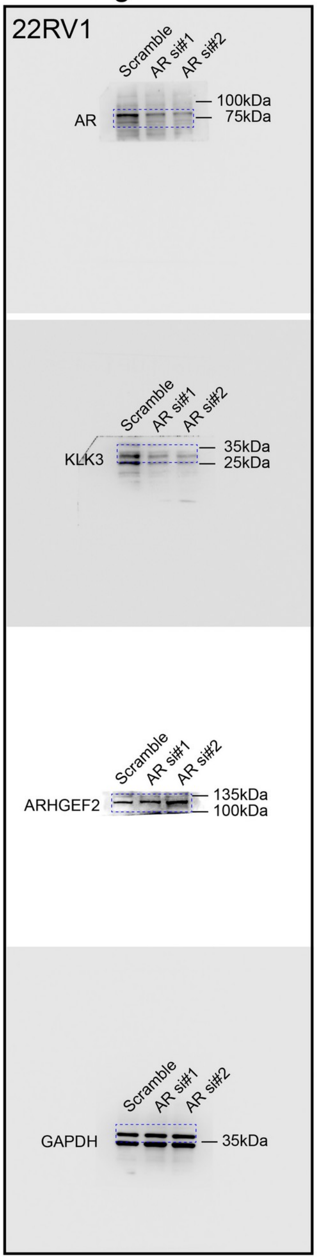

Figure 4A/D

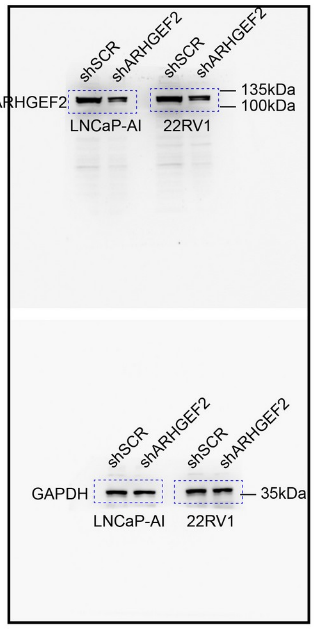

Figure 4G

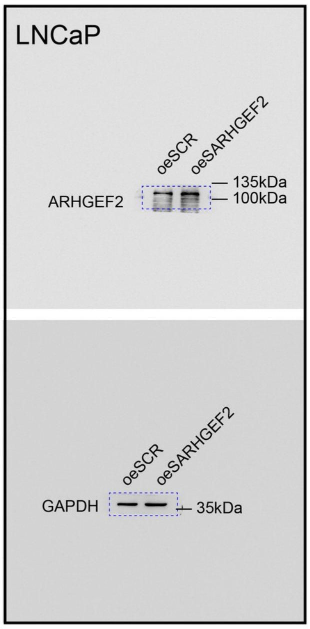

Figure 4B

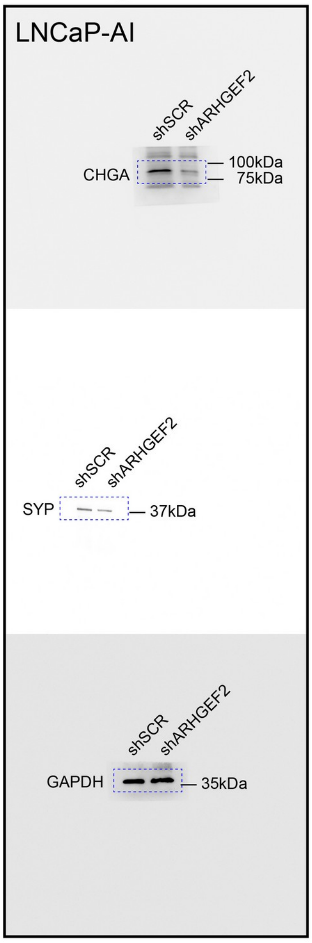

Figure 4E

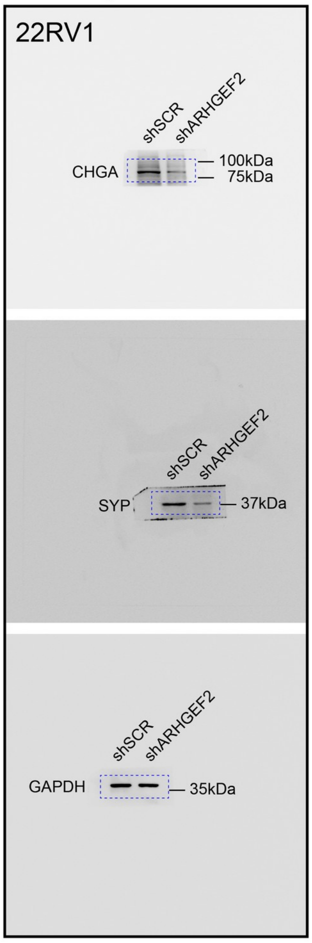

Figure 4H

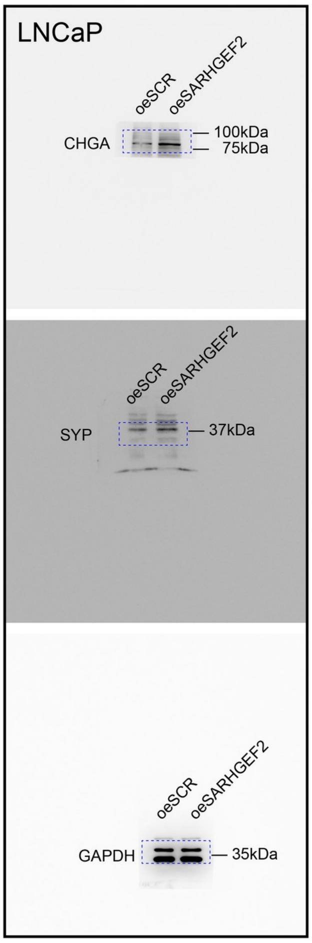

Figure 5D Figure 5E Figure 5F Figure 5G

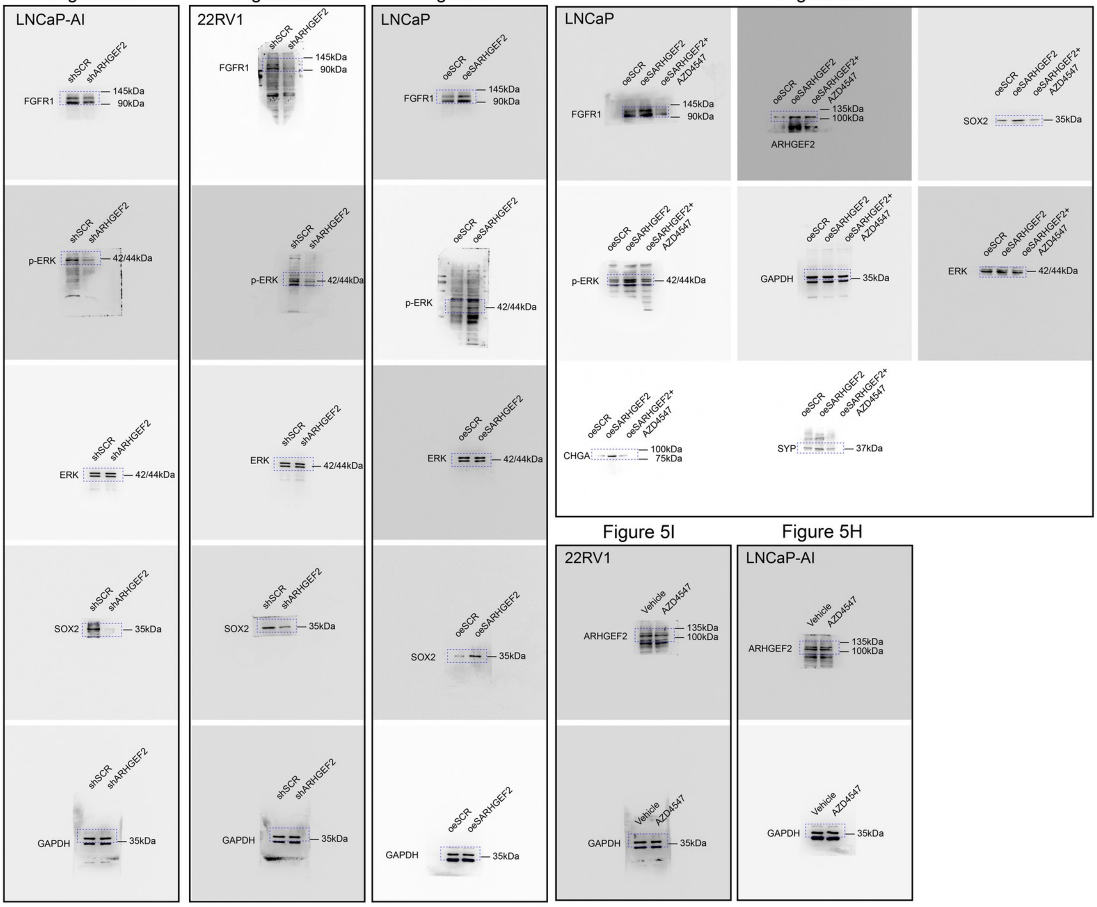

Figure S2D Figure S2E Figure S6

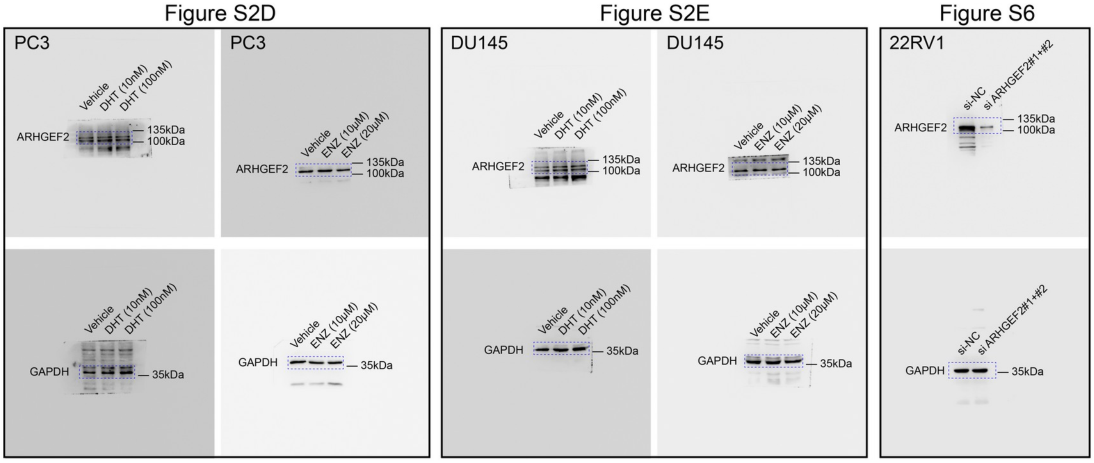

Supplement: Supplementary file 2 [file 41419_2022_5366_MOESM3_ESM.docx]
